# CD300E^+^ macrophages facilitate liver regeneration after splenectomy in decompensated cirrhotic patients

**DOI:** 10.1038/s12276-024-01371-3

**Published:** 2025-01-01

**Authors:** Tao Yang, Yuan Zhang, Chujun Duan, Hui Liu, Dong Wang, Qingshan Liang, Xiao Chen, Jingchang Ma, Kun Cheng, Yong Chen, Ran Zhuang, Jikai Yin

**Affiliations:** 1 Department of General Surgery, Tangdu Hospital of the Air Force Medical University, 569 Xin Si Road, Xi’an, 710038 Shaanxi China; 2 Department of Immunology, Air Force Medical University, 169 West Changle Road, Xi’an, 710032 Shaanxi China; 3 Department of Hepatobiliary Surgery, Xijing Hospital of the Air Force Medical University, 15 West Changle Road, Xi’an, 710032 Shaanxi China

**Keywords:** Liver cirrhosis, Cell proliferation

## Abstract

Liver cirrhosis is prognostically associated with poor life expectancy owing to subsequent liver failure. Thus, understanding liver regeneration processes during cirrhotic injury is highly important. This study explored the role of macrophage heterogeneity in liver regeneration following splenectomy. We collected detailed clinical information from 54 patients with decompensated cirrhosis before and after splenectomy. Obvious liver regeneration was observed after splenectomy in cirrhotic patients. Single-cell RNA sequencing (scRNA-seq) was performed on three paired liver tissues from patients before and after surgery to explore the immune microenvironment map and the characteristics of liver regeneration-associated macrophages (RAMs). scRNA-seq analysis revealed that the composition of hepatic immune cells changed after splenectomy; among these changes, the proportion of CD300E^+^ RAMs significantly increased after surgery, and high expression levels of functional genes associated with cell proliferation promoted liver regeneration. Moreover, a mouse model of carbon tetrachloride-induced cirrhosis and a coculture system consisting of primary bone marrow-derived macrophages and hepatocytes were established for validation. We observed a similar phenomenon of liver regeneration in cirrhotic mice and further confirmed that CD300E^+^ monocyte-derived macrophages facilitated hepatocyte NAD^+^ synthesis via the secretion of NAMPT, which subsequently promoted hepatocyte proliferation. This study characterized the hepatic immune microenvironment in patients with cirrhosis following splenectomy. Our findings demonstrated that CD300E^+^ macrophages play a crucial role in remodeling the hepatic immune microenvironment after splenectomy, thereby promoting liver regeneration in patients with decompensated cirrhosis. CD300E^+^ macrophages are anticipated to emerge as a novel therapeutic strategy for the treatment of liver cirrhosis.

## CD300E+ macrophages drive liver regeneration after splenectomy

Chronic liver disease can progress to cirrhosis, a condition where the liver is severely scarred and its ability to regenerate is impaired, leading to serious health problems. They examined liver tissues from 54 patients with cirrhotic portal hypertension, a condition where high blood pressure occurs in the veins of the liver, before and after splenectomy. Using single-cell RNA sequencing, a technique to study individual cell types, they observed changes in immune cells. The study showed that removing the spleen changes the liver’s immune environment, increasing liver size and function. CD300E^+^ macrophages, a type of immune cell, were found to help liver cells grow by releasing NAMPT, a protein important for energy use. The findings suggest splenectomy could support liver healing by altering immune cells, offering new treatment possibilities for chronic liver damage.

This summary was initially drafted using artificial intelligence, then revised and fact-checked by the author.

## Introduction

Chronic liver disease causes continuous liver injury, which eventually leads to cirrhosis. Although the liver has a strong ability to clear xenobiotics, repair damage, and regenerate new cells to remodel the liver volume, cirrhosis caused by persistent liver injury has adverse effects on liver regeneration that cause portal hypertension and liver failure, which seriously affect the prognosis of patients. Liver transplantation is the only widely accepted etiologic treatment for this condition, but it is difficult to apply routinely because of its high cost and insufficient number of donors^1^.

Splenectomy is a clinical option for treating hypersplenism secondary to portal hypertension^2^. Clinicians have reported that splenectomy can improve coagulation function, liver function, the tumor immune response, and long-term survival in patients with portal hypertension^3–6^. Our previous observations indicated that patients with portal hypertension benefit from increased liver volume in the months after splenectomy^7^. These findings suggest that the spleen may be involved in regulating hepatic immune homeostasis, thereby affecting liver regeneration. Although the spleen is the largest secondary lymphoid organ in the body, its effects on liver regeneration are less studied and have not been fully recognized.

In this study, we collected clinical data and liver tissues from patients with cirrhotic portal hypertension to analyze the pathophysiological features of the liver before and after splenectomy. Single-cell RNA sequencing (scRNA-seq) technology was first applied to reveal alterations in the immune microenvironment of the liver at the cellular level after splenectomy. Our results increase the understanding of immune cell changes during liver regeneration and provide a novel intervention target for further exploration of cirrhosis-related therapeutic strategies.

## Materials and methods

### Patients

The clinical data of patients diagnosed with liver cirrhosis and portal hypertension at the Department of General Surgery of the Second Affiliated Hospital of the Air Force Medical University were collected from March 2016 to March 2022. The patient underwent laparoscopic splenectomy with pericardial devascularization. Patients were assessed via thin-slice abdominal computerized tomography (CT), and blood tests were performed before and 6 months after splenectomy. Hepatic venous pressure gradient (HVPG) measurements were performed before surgery in all patients, and postoperative HVPG data were missing for 25 patients. The inclusion and exclusion criteria are listed in the Supplementary Materials and Methods. Liver volume was measured via Myrian^®^ Studio software (Intrasense, Montpellier, France). Ethical approval for this study (No. K202001-07) was provided by the Medical Ethics Committee of Tangdu Hospital, Air Force Medical University. All the research was conducted in accordance with the Declaration of Helsinki and Istanbul. Written consent was given by all the subjects.

### Clinical liver sample collection

Approximately 0.5 cm^3^ of liver tissue was obtained during surgery. Some tissue samples were fixed with 4% paraformaldehyde (Servicebio, G1101-500ML), and the remaining samples were immediately digested in a gentleMACS™ C tube (Miltenyi Biotec, 130-093-237) preloaded with 4 ml of digestive enzymes for subsequent tissue dissociation. Ultrasound-guided percutaneous liver biopsy was performed 3 months after surgery. Some tissues were fixed, and the remaining tissues were immediately placed into a C tube preloaded with 2 ml of tissue digestive enzymes. All the samples were treated in accordance with ethical and legal standards, and patient anonymity was maintained.

### Single-cell sample preparation and scRNA-seq analysis

The samples were digested, and a single-cell suspension was prepared. Following quality control, a sequencing library was constructed, and high-throughput sequencing was performed. Cell Ranger was used for quality control of the original data, and Harmony^8^ was used for data consolidation and batch effect correction. The cells were clustered via principal component analysis^9^, and the results were visualized via *t*-distributed stochastic neighbor embedding (*t*-SNE) and Uniform Manifold Approximation and Projection (UMAP). SingleR^10^ was used for cell annotation and combined with manual annotation to identify cell types. Differentially expressed genes (DEGs) enriched in Gene Ontology (GO)^11^ and Kyoto Encyclopedia of Genes and Genomes (KEGG)^12^ were assessed, and their functions and pathways were obtained. Velocyto^13^ and Monocle^14^ were used for RNA velocity and pseudotime trajectory analyses, respectively, to construct the single-cell differentiation trajectory. Cell interaction analysis was performed via CellPhoneDB^15^ and NicheNet^16^. See the Supporting Information for additional details.

### Hematoxylin‒eosin (H&E) and Masson trichrome staining

H&E staining was performed on 5 μm paraffin sections via standard protocols. ImageJ software was used to obtain cell counts and conduct area measurements. Masson’s trichrome staining was performed via a commercial kit (Servicebio, G1006-100ML). Images were obtained via the Invitrogen EVOS M7000 Imaging System. See the Supplementary Materials and Methods for additional details.

### Fluorescent multiplex immunohistochemistry (mIHC)

Fluorescent mIHC was performed via tyramide signal amplification (TSA) technology^17^. Briefly, the tissue sections were dewaxed in water, antigens were retrieved, and primary and secondary antibody treatments were performed. Fluorescence images were obtained using a microscope (Nikon Eclipse C1, Tokyo, Japan). Detailed information is provided in the Supplementary Materials and Methods.

### Animals

Male C57BL/6 mice, 6–8 weeks old, were purchased from the Laboratory Animal Center of the Air Force Military Medical University. The mice were housed in a specific pathogen-free facility at 21 ± 2 °C with a 12 h dark/light cycle. The mice were allowed to adapt for 1 week before the experiment and were allowed to drink and eat freely. The mice were randomly allocated into three groups (*n* = 6/group): the liver cirrhosis (LC), LC + splenectomy (sp), and LC + sham operation (sham) groups. Modeling approach: Carbon tetrachloride (CCl4) (Macklin, C805325) was diluted in corn oil (Macklin, C805618-500 ml) at a ratio of 1:4 (concentration of 20% CCl4) and injected intraperitoneally at 1 μl/g of CCl4 twice weekly for a total of 12 weeks^18^. On the first day of the 13th week, splenectomy was performed under isoflurane anesthesia; for the sham surgery the spleen was exposed and the abdominal cavity was closed without excision, and CCl4 injection was continued for 2 weeks after the operation. The mice were sacrificed on the first day of the 15th week, the hearts were perfused with precooled phosphate-buffered saline (PBS) (Servicebio, G4202-500ML), and the livers were harvested for subsequent experiments. All animal experiments were approved by the Ethics Committee of the Air Force Medical University and performed in accordance with the experimental protocol for animal handling of the Air Force Medical University.

### Splenectomy

The abdominal surgical area of each mouse was shaved and sterilized prior to surgery. After anesthesia with isoflurane, the abdomen was disinfected with iodophor, and a longitudinal incision of approximately 1 cm in length was made in the midline of the abdomen. The spleen was exposed and lifted on the side of the greater curvature of the stomach, and the perisplenic ligaments and blood vessels were dissected by electrocoagulation along the medial edge of the spleen to complete the splenectomy. After the procedure, the mice were intraperitoneally administered 1 ml of warm normal saline to prevent dehydration, and the abdominal cavity was closed and rewarmed to allow mice to wake a warm box before being released back to the cage.

### Immunofluorescence

Immunofluorescence staining of paraffin sections and cell-climbing slices was performed via standard protocols. The detailed procedure is described in the Supplementary Materials and Methods. Immunofluorescence images were acquired using a fluorescence microscope (EVOS M7000, Invitrogen).

### Isolation of hepatic mononuclear cells

In reference to a previous methodology^19^, the liver was excised, homogenized and passed through a 70 μm cell strainer (Corning, 352350) using a grinding rod. The liver cells were suspended in PBS and centrifuged at 50 × *g* for 5 min, the supernatant was collected and centrifuged at 500 × *g* for 10 min at 4 °C, and the cells were resuspended in 40% Percoll (Yeasen, 40501ES60). The cells were then gently overlaid onto a 70% Percoll gradient, and then centrifuged at 750 × *g* for 30 min at room temperature. Hepatic mononuclear cells were collected from the cloudy layer, washed twice in PBS, counted, and resuspended in PBS for flow cytometry.

### Isolation and induction of mouse bone marrow monocytes

Monocytes were isolated according to the manufacturer’s instructions for the mouse bone marrow monocyte isolation kit (TBD, TBD2013DM). The detailed procedure is described in the Supplementary Materials and Methods. The collected cells were seeded in 6-well plates and cultured in DMEM/F12 (Gibco, 11320032) supplemented with 10% fetal bovine serum (FBS) (Yeasen, 40130ES76), penicillin‒streptomycin solution (Mishu, MI00614) and 20 ng/mL macrophage colony-stimulating factor (M-CSF) (MedChemExpress, HY-P7085)^20^. The cells that had adhered after 6 days of incubation were considered bone marrow-derived macrophages (BMDMs). The purity of the BMDMs was determined via flow cytometry analyses^21^.

### Cell transfection

To overexpress CD300E in BMDMs, a *Cd300e* overexpression plasmid vector tagged with EGFP fluorescence was constructed by Tsingke Biotech Co. Ltd. Cells were transfected with Lipofectamine 3000 Transfection Reagent (Invitrogen, L3000015) according to the manufacturer’s instructions. GFP fluorescence images were acquired using a fluorescence microscope (EVOS FL, Thermo Fisher Scientific).

### Isolation of mouse primary hepatocytes

Mouse primary hepatocytes were isolated and cultured as reported in previous studies^22^. Briefly, the mice were anesthetized via an intraperitoneal injection of 3% pentobarbital sodium (Sigma, P3761). Primary hepatocytes were isolated via a two-step collagenase perfusion protocol, resuspended in complete medium (DMEM/F12, 10% FBS and 1% penicillin/streptomycin), and seeded in Transwell upper chambers (Corning, 3412); partial cells were used to create cell climbing slices (WHB scientific, WHB-6-CS) for subsequent experiments.

### Transwell coculture system

Mouse primary BMDMs (lower chamber) and hepatocytes (upper chamber) were cocultured using a Transwell system (Corning, 3412). The experiment included four groups: negative control (NC), vector, CD300E-overexpressing (OE), and CD300E OE + FK866 (Nampt inhibitor) (MedChemExpress, HY-50876). The cells were collected after 48 h of coculture.

### Flow cytometry analysis

A single-cell suspension of hepatic mononuclear cells was washed in PBS containing 1% BSA. The surfaces of the cells were stained with fluorochrome-conjugated monoclonal antibodies for 30 min on ice. Antibody information is provided in the Supplementary Materials and Methods. The samples were acquired on a SONY SA3800 Spectral Analyzer, and the data were analyzed via FlowJo software (version 7.6.5 TreeStar).

### Western blot

Transwell upper chamber cell lysates were prepared by adding 200 μL of RIPA lysis buffer (Genstar, E121-01) mixed with protease inhibitor (Epizyme Biotech, GRF101) (100:1) on an ice plate. Hepatocytes were scraped from the upper chamber and lysed thoroughly. The protein concentration was determined using a BCA protein assay kit (Genatar, E162-01). The cell lysates were separated by 12.5% SDS‒PAGE, and the proteins were transferred to PVDF membranes (Millipore, IPVH00010). The primary antibody PCNA was incubated overnight at 4 °C, and the secondary antibody HRP-conjugated AffiniPure goat anti-rabbit IgG (H + L) (Proteintech Group, SA00001-2) was incubated at room temperature for 1 h. An enhanced chemiluminescence (ECL) kit (Mishu, MI00607) was used for development. The membranes were stripped with fast stripping buffer (Epizyme Biotech, PS107) before reblotting, and an anti-β-actin antibody (Servicebio, ZB15001-HRP-100) was incubated at room temperature for 3 h. An ECL kit was used for development. Imaging was performed using a ChemiDoc XRS+ System (Bio-Rad).

### ELISAs

The cytokine NAMPT was detected in the medium using ELISA kits (Fankew, F30252-A) according to the manufacturer’s instructions. The absorbance at 450 nm (OD450) was measured using a Multiskan FC microplate reader (Thermo Fisher).

### Nicotinamide adenine dinucleotide (NAD^+^) assays

The cells were collected with NAD^+^/NADH extraction buffer. The intracellular NAD^+^ content was determined using an NAD^+^/NADH assay kit with WST-8 (Beyotime, S0175) according to the manufacturer’s protocol. The resulting values were normalized to the total cell number.

### ATP assays

The cells were then lysed using lysis buffer associated with the Enhanced ATP Assay Kit (Beyotime, S0027), and the ATP content was determined according to the manufacturer’s instructions. The resulting values were normalized to the total cell number.

### Statistical analysis

Continuous clinical data that adhered to a normal distribution are expressed as the means ± standard deviations (SDs), and experimental data are expressed as the means ± standard errors of the means (SEMs). Nonnormal variables are expressed as medians (interquartile spacing) and were compared via the Mann‒Whitney *U* test. Paired or unpaired Student’s *t* tests were used to compare data between two groups. One-way analysis of variance (ANOVA) with post hoc Tukey’s multiple comparison test was performed to compare three or more groups. The Wald test was used to compare the proportions of subgroup cells before and after surgery^23^. SPSS (version 21.0, IBM Corp, Armonk, NY, USA) was used for the statistical analyses, and *p* < 0.05 was considered statistically significant. Graphs were created via GraphPad Prism (version 9.0, GraphPad Software, Boston, Massachusetts, USA).

## Results

### Liver regeneration after splenectomy in patients with liver cirrhosis

**Fig. 1:**
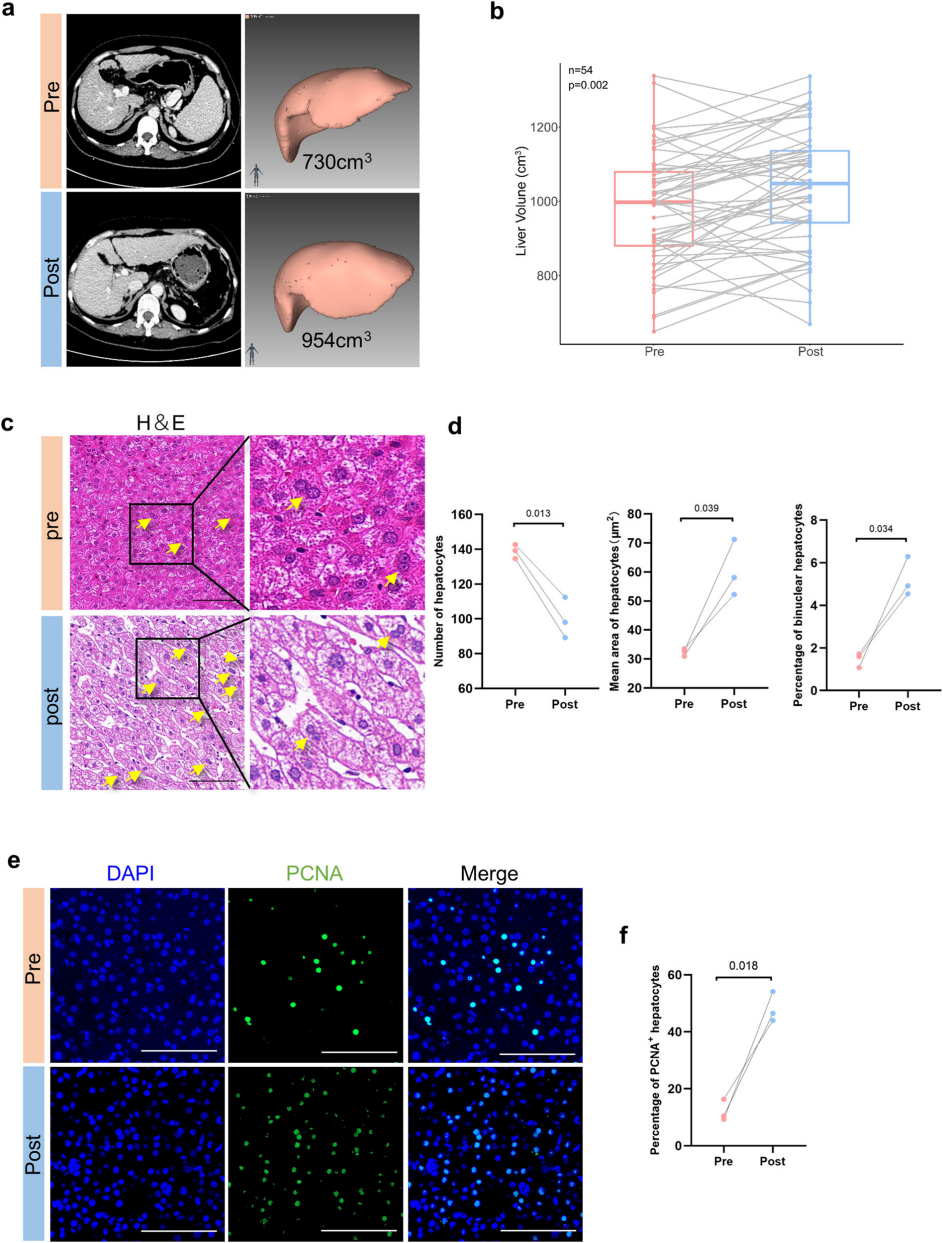
Liver regeneration increased after splenectomy. **a** Pre- and postoperative liver CT (planes of the left and right bifurcations of the portal vein) and liver volume measurements. **b** Box plot showing that 40 of 54 patients had increased liver volume after surgery, and 14 had decreased liver volume. **c** Masson and H&E staining images of liver tissues: the yellow arrow indicates binuclear hepatocytes, and the local enlargement is shown to the right of the black box. **d** The number of hepatocytes, the proportion of binuclear cells, and the average area of hepatocytes were statistically analyzed at the same magnification and within the same area of the visual field. **e**, **f** Representative immunofluorescence images: DAPI (blue), PCNA (green), and hepatocyte proliferation were quantified by counting the PCNA-positive cells. Five randomly selected microscopic fields were captured per sample and quantified via ImageJ. Scale bars, 100 μm. The data represent the means ± SEMs. Statistical significance was determined via paired *t-*tests. Pre preoperation, Post postoperation.

A total of 54 patients with liver cirrhosis who underwent splenectomy were enrolled according to the inclusion and exclusion criteria, as shown in Supplementary Table 1. The clinical characteristics of all the subjects before and after the surgery indicated that in addition to significantly increased white blood cell (WBC) and platelet (PLT) counts after splenectomy, liver function and coagulation-related indicators, such as model for end-stage liver disease (MELD) scores, albumin levels, the prothrombin time (PT), and the international normalized ratio (INR), were also markedly improved (Table 1). The liver volume was measured via thin-slice CT (Fig. 1a), which revealed that 74% of the patients (40 patients) had significantly enlarged liver volumes postoperatively (Fig. 1b); this increase indicated regeneration of the liver. We then collected three pairs of liver tissues from the patients before and after the surgery (Supplementary Table 2). H&E staining of the liver sections revealed a significantly decreased quantity of hepatocytes postsurgery, with enlarged hepatocyte volumes and increased numbers of binuclear hepatocytes (Fig. 1c, d). Immunofluorescence staining for PCNA also revealed that the percentage of positive hepatocytes significantly increased after splenectomy, which suggested that cell proliferation was ongoing (Fig. 1e, f). Hence, liver regeneration occurs after splenectomy in patients with cirrhosis.

**Table 1:** Comparison of preoperative and postoperative indices across all subjects.

Parameters	Total (*n* = 54)		*p*
	Preoperation	Postoperation	
Liver volume (cm^3^)	995.78 ± 212.45	1054.26 ± 218.94	**0.001**
HVPG (mmHg)^a^	16.03 ± 5.28	12.79 ± 6.82	0.060
MELD score	10.15 ± 2.16	8.74 ± 2.19	**<0.001**
Child‒Pugh score	6 (6, 7)	6 (6, 7)	0.116
Child‒Pugh classification			**0.029**
A	28	39	
B	26	15	
ALT (U/L)	26 (22.75, 40.50)	32.50 (26.75, 39.50)	0.089
AST (U/L)	27.50 (20.00, 37.25)	39 (28, 43.25)	**0.003**
ALB (g/L)	38.01 ± 4.35	40.25 ± 5.65	**0.019**
TBIL (μmol/L)	23.88 ± 10.39	21.3 ± 12.95	0.156
RBC (×10^12^/L)	3.42 ± 0.51	3.93 ± 0.61	**<0.001**
WBC (×10^9^/L)	1.82 ± 0.96	6.98 ± 2.91	**<0.001**
PLT (×10^9^/L)	47.35 ± 25.14	228.7 ± 84.28	**<0.001**
PT (s)	14.69 ± 1.59	13.06 ± 1.56	**<0.001**
INR	1.26 ± 0.14	1.13 ± 0.15	**<0.001**

*HVPG* hepatic venous pressure gradient, *MELD* model for end-stage liver disease score, *ALT* alanine aminotransferase, *AST* aspartate amino transferase, *ALB* albumin, *TBIL* total bilirubin, *RBC* red blood cell count, *WBC* white blood cell count, *PLT* platelet count, *PT* prothrombin time, *INR* international normalized ratio.

^a^29 patients were matched.

Note: Bolded values are indicative of a *P* value of less than 0.05.

### Immune microenvironment profile of the cirrhotic liver before and after splenectomy at the single-cell level

**Fig. 2:**
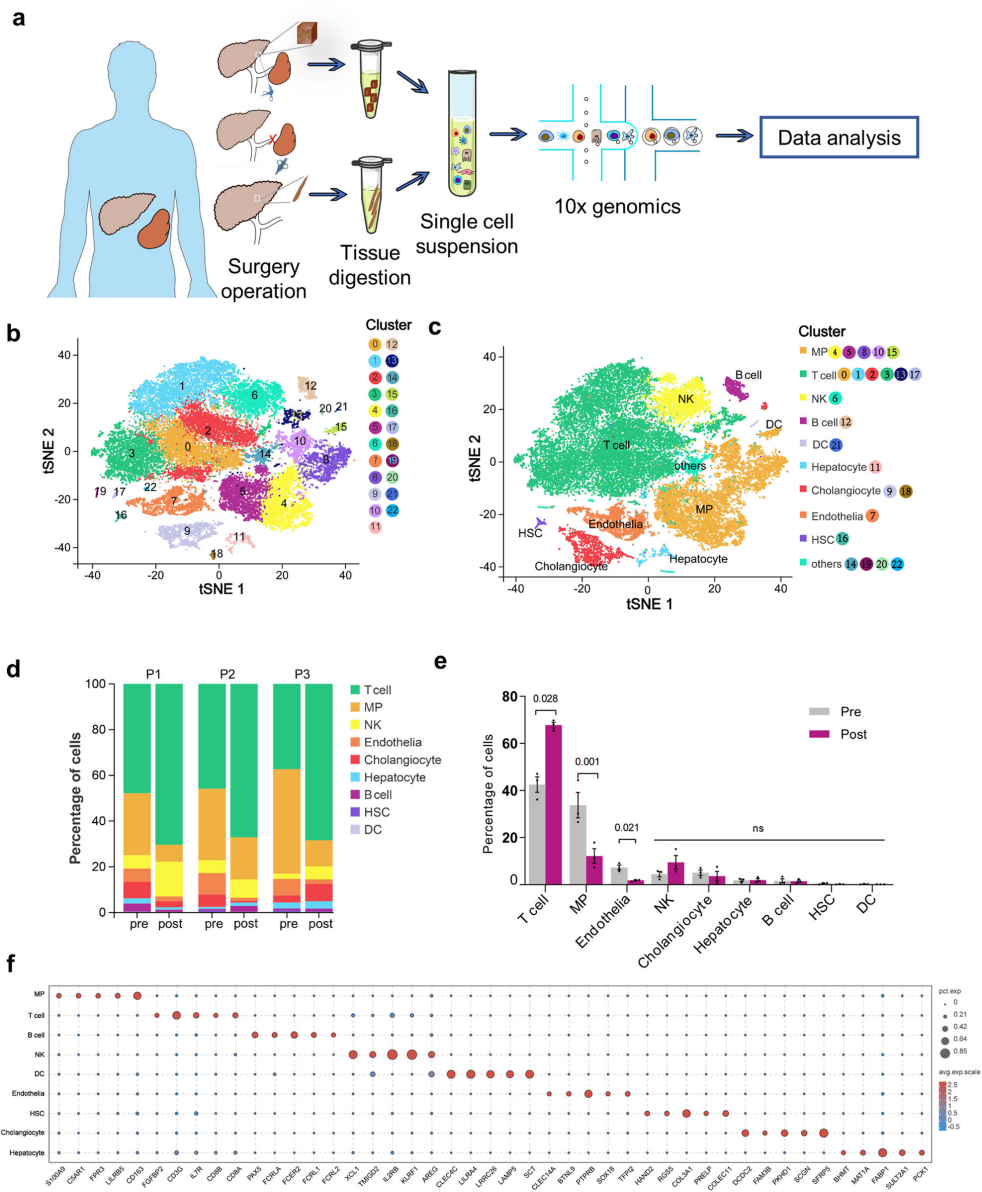
ScRNA-seq analysis revealed postoperative changes in the hepatic immune microenvironment. **a** Flowchart of the scRNA-seq experiment. **b** t-SNE plot representing 23 subsets of liver cells identified after dimension reduction and clustering, which are labeled with different colors. **c** t-SNE plot showing nine cell subsets after annotation. Others refers to undefined cells. **d**, **e** Bar chart showing the proportions of the nine cell subsets in each sample before and after surgery. Statistical analysis of the percentage of cells in all three samples before and after surgery. Statistical significance was determined via the Wald test. **f** Bubble chart showing the expression of the top five upregulated genes in each cell population. The bubble color represents the average expression (avg.exp.) of the gene, and the bubble size represents the percentage of cells expressing (pct exp.) the gene. P1(2,3), patient 1(2,3). Pre preoperation, Post postoperation.

scRNA-seq was performed on six liver tissue samples from three patients with cirrhosis before and after surgery (Fig. 2a). A total of 36,498 liver cells, including 26,077 cells before surgery and 10,421 cells after surgery, were obtained after cell filtration and quality screening. Cell classification and subpopulation identification were conducted via Seurat to construct the liver immune microenvironment profile. We identified 23 cell subsets after dimension reduction and unsupervised clustering (Fig. 2b), which were annotated into nine cell clusters based on known cell markers (Supplementary Fig. 1a), including mononuclear phagocytes (MPs), T cells, natural killer cells (NKs), B cells, dendritic cells (DCs), hepatocytes, cholangiocytes, endothelial cells, hepatic stellate cells (HSCs)^23–25^ and some undefined cells (Fig. 2c). The cell clustering results before and after surgery were consistent (Supplementary Fig. 1b), with highly expressed marker genes in their respective cell subsets (Supplementary Fig. 1c). In addition, all the cells expressed high levels of housekeeping genes (*ACTB*, *B2M*, *GAPDH*, *HSP90AB1*, *PPIA*, *RPLP0*, *RPLP1*, and *PTPRC*)^26,27^, further highlighting the accuracy of the data (Supplementary Fig. 1d).

Through clustering analysis, we found that the proportion of T cells significantly increased (45.05% vs. 69.48%) and that the proportion of MPs significantly decreased (32.34% vs. 10.08%) after splenectomy, as depicted in Fig. 2d, e. Further analysis of all upregulated genes in different cell types (Supplementary Fig. 1e) revealed the top five genes that were expressed specifically in each cell population (Fig. 2f). These results suggest that the hepatic immune microenvironment significantly changes in cirrhotic patients following splenectomy.

### Heterogeneity and potential differentiation of hepatic macrophages in cirrhotic livers after splenectomy

**Fig. 3:**
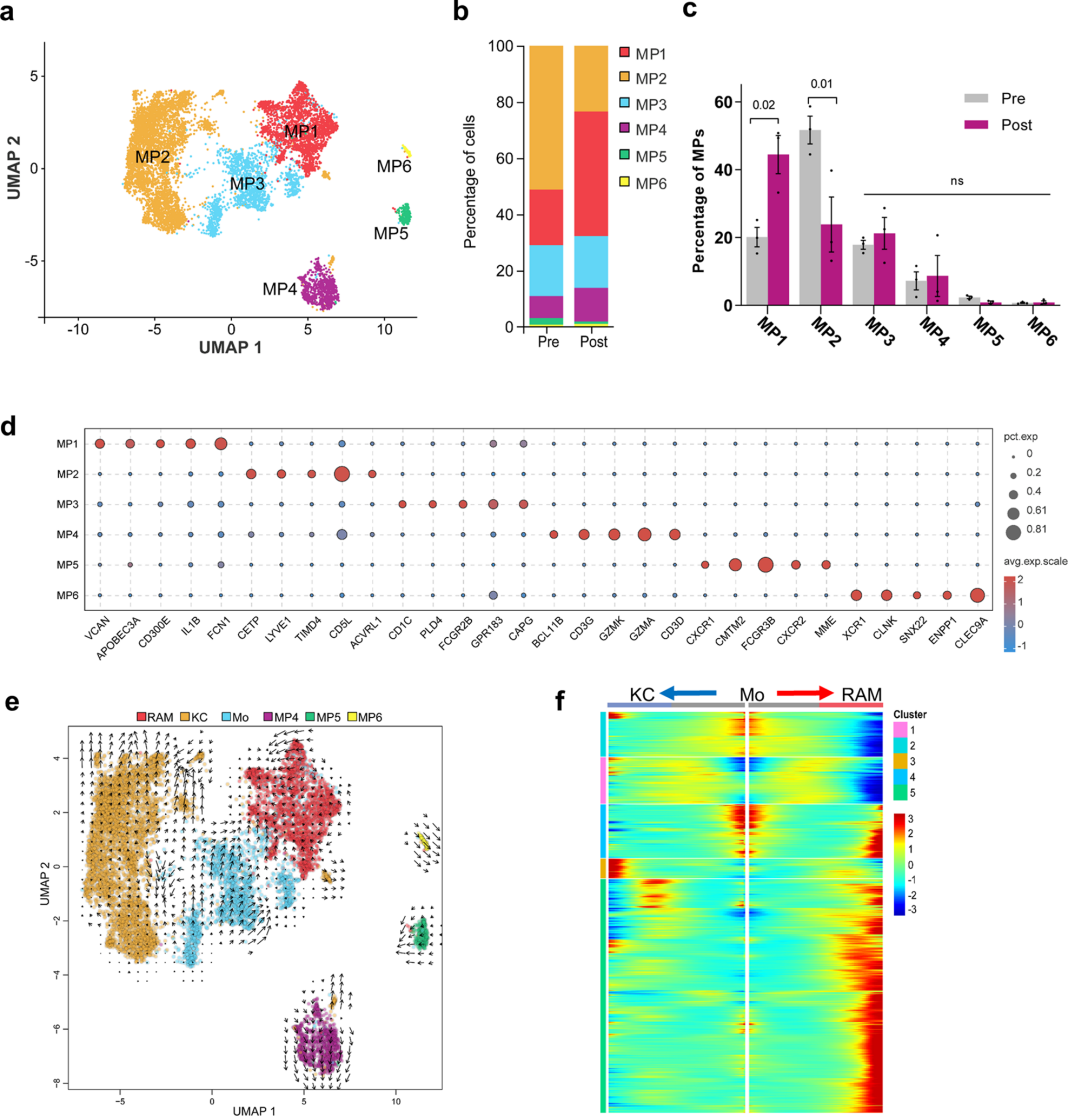
Mononuclear phagocyte (MP) subset analysis. **a** UMAP represents the six MP subsets identified after the cells were regrouped. **b**, **c** Bar chart showing the percentages of MP subsets before and after surgery. Statistical analysis of the proportion of each MP subset before and after surgery. Statistical significance was determined via the Wald test. **d** Bubble chart showing the expression of the top five upregulated genes in each MP subset. The bubble color represents the average expression (avg.exp.) of the gene, and the bubble size represents the percentage of the cells expressing (pct exp.) the gene. **e** Mapping of RNA velocity to UMAP showing the differentiation trend among MP subsets, with different colors representing each subset and arrows representing the direction of differentiation. **f** Heatmap showing the expression of five clusters of DEGs (at pseudotime branching node 3) via pseudotime trajectory analysis. The *x*-axis represents pseudotime points (with a gradual increase from the middle to both sides), the *y*-axis represents branch DEGs (not specifically shown), and the red to blue represents the expression abundance of genes from high to low at the pseudotime points. The clusters represent classes of genes with similar branching expression trends. RAM regeneration-associated macrophage, KC Kupffer cell, Mo monocyte.

Macrophages, not T cells, are the immune effector cells involved in liver regeneration. Previous studies have shown that macrophage heterogeneity plays an important role in the progression of liver regeneration^28,29^; however, the role it plays in the process of liver regeneration following splenectomy in cirrhotic patients remains unclear. To further study the heterogeneity of liver macrophages after splenectomy, we clustered the MPs into six subsets, which were annotated as MP1-6 (Fig. 3a). We found that the proportion of MP1 cells increased significantly (19.78% vs. 44.32%) and that the proportion of MP2 cells decreased significantly (51.22% vs. 23.41%) after splenectomy (Fig. 3b, c). We also analyzed the upregulated genes (Supplementary Fig. 2a) and displayed the expression profile of the top five significantly upregulated genes in each MP subset (Fig. 3d). Among the six subsets, the gene expression profile of MP2 was consistent with that previously reported for the resident liver macrophages, Kupffer cells (KCs)^30^, as shown in Supplementary Fig. 2b. MP3 exhibited increased expression of monocyte-associated markers (such as *CD1C*, *PLD4*, *GPR183*, and *CAPG*^31–33^). Because the proportion of MP1s increased significantly after splenectomy, we labeled them regeneration-associated macrophages (RAMs).

Macrophage heterogeneity manifests as cell polarization and phenotypic transformation^34,35^. RNA velocity and pseudotime trajectory analyses were performed on the macrophage subpopulations to evaluate the presumed transforming relationship between RAM and other MPs. The RNA velocity results revealed that monocytes tended to differentiate into RAMs (Fig. 3e). Pseudotime trajectory analysis of RAMs, KCs and monocytes was conducted via Monocle to obtain the distributions of the corresponding subsets and distinct differentiation statuses. The results revealed that monocytes were in the early stage of differentiation (State 1), whereas RAMs (State 7) and KCs (State 6) were at the end stage of differentiation (Supplementary Fig. 3a–c). Pseudotime trajectory analysis of the branch (Node 3)-dependent DEGs related to certain differentiation fates revealed five gene clusters, with various gene expression characteristics in each cluster (Fig. 3f). Hence, different gene expression trends in the monocyte subset could lead to distinct cell differentiation into RAMs or KCs. Through GO analysis of the DEGs in each cluster, we found that these genes were enriched in biological processes related to the regulation of macrophage polarization, such as oxidative phosphorylation, cell metabolism, and cell activation. (Supplementary Fig. 3d). We visualized the genes with the most significant differences in each cluster, namely, *C1QC*, *RPS27A*, *B2M*, *MALAT1*, and *MT-ND1* (Supplementary Fig. 3e), which may have promoted the development and differentiation of these subsets. Understanding the potential differentiation relationships among RAMs will establish a crucial foundation for subsequent investigations into the origin of RAMs.

### CD300E^+^ RAMs promote the proliferation of liver parenchymal cells

**Fig. 4:**
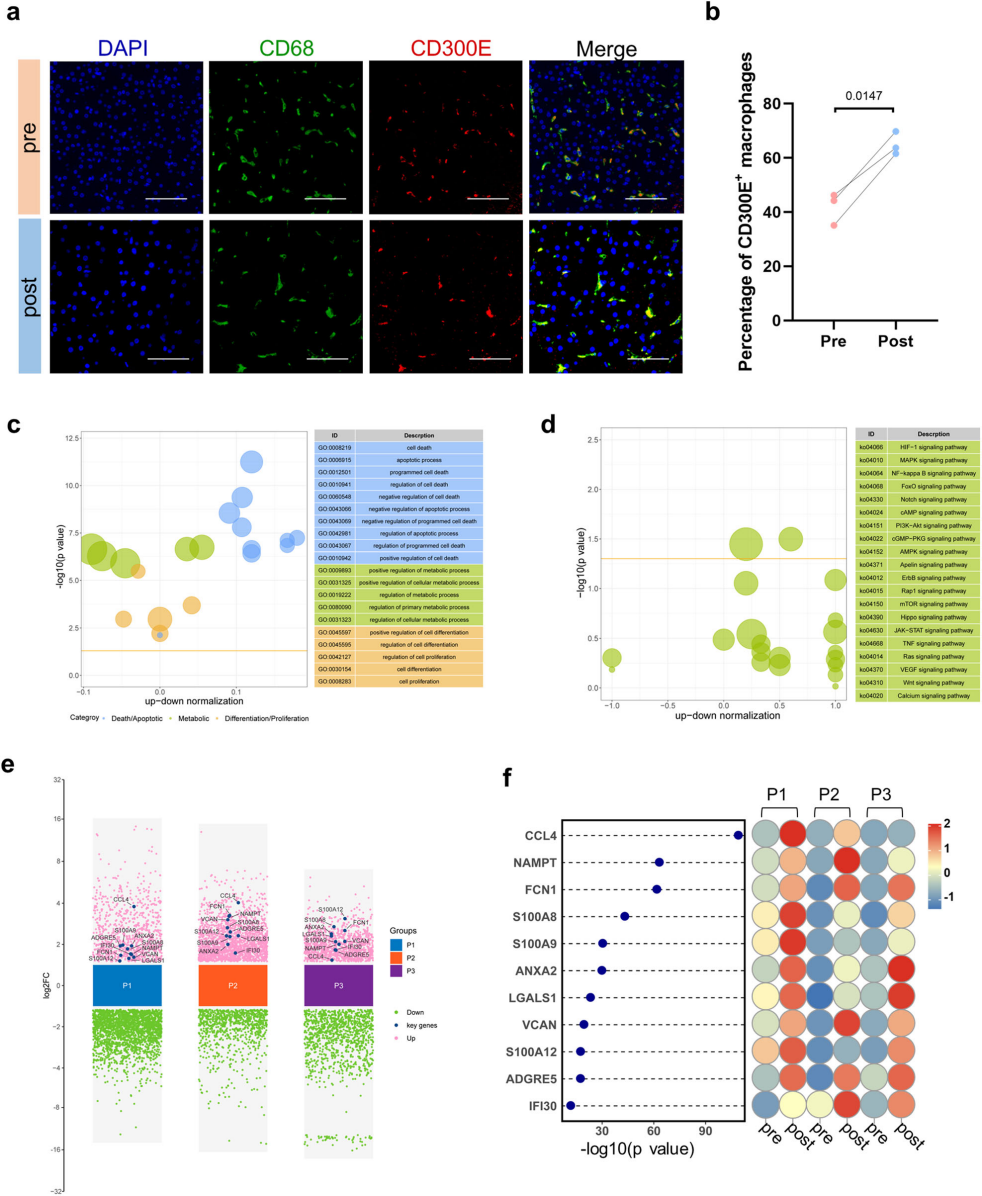
Functional analysis of CD300E^+^ RAM. **a**, **b** Immunofluorescence staining showing the coexpression of CD300E (red) and CD68 (green) in liver tissues before and after surgery; DAPI (blue) was used. Five randomly selected microscopic fields were captured per sample and quantified via ImageJ. Scale bars, 100 μm. The data represent the means ± SEMs. Statistical significance was determined via paired *t-*tests. Pre preoperation, post postoperation. **c** Bubble chart showing the enriched GO terms of the DEGs in the CD300E^+^ RAMs. The bubble size represents the number of target genes enriched in the current term, and the yellow line represents the threshold of a *p* value = 0.05. The filtered term is shown on the right. **d** Bubble chart showing the enriched KEGG pathways of the DEGs in CD300E^+^ RAMs. The bubble size represents the number of target genes enriched by the current pathway, the yellow line represents the threshold of *p* value = 0.05, and the filtered pathway is shown on the right. **e** Multigroup difference scatter plot showing the significant DEGs in each sample group between pre- and postoperative data. Eleven key genes were labeled after screening, and the *y*-axis represents the log2-fold change (FC). **f** Heatmap showing the differences in the expression of 11 upregulated DEGs in all three sample groups before and after surgery (right). The 11 genes were sorted by −log10 (*p* value) (left).

The proportion of RAMs significantly increased after splenectomy, which suggested that they may exert positive effects during liver regeneration. Therefore, we selected CD300E, the cell surface protein with the most significant increase after splenectomy (Supplementary Fig. 4a), to specifically label this population. Immunofluorescence staining further verified the costaining of CD300E with the macrophage marker CD68 in pre- and postoperative liver tissues, with the percentage of CD300E^+^ macrophages significantly increased post-operation (Fig. 4a, b). Next, we investigated the function of the CD300E^+^ RAM subgroup via DEG analysis (Supplementary Fig. 4b). The intersection of DEGs of the RAM subset in all three groups of samples revealed 116 commonly upregulated DEGs and 166 commonly downregulated DEGs (Supplementary Fig. 4c). GO functional enrichment and KEGG pathway enrichment analyses of the DEGs revealed that these genes were enriched in biological processes related to death, apoptosis, metabolism, differentiation, and proliferation and pathways related to HIF-1 and MAPK signaling (Fig. 4c, d). Macrophages are located in the hepatic sinuses and act on hepatic parenchymal cells by secreting cytokines. From the above 116 upregulated DEGs, we screened 11 genes that encode secreted proteins via the GeneCards public database (www.genecards.org) and found that they were all significantly upregulated after surgery, especially *CCL4* and *NAMPT* (Fig. 4e, f and Supplementary Fig. 4d).

### Communication between CD300E^+^ RAMs and hepatic parenchymal cells

**Fig. 5:**
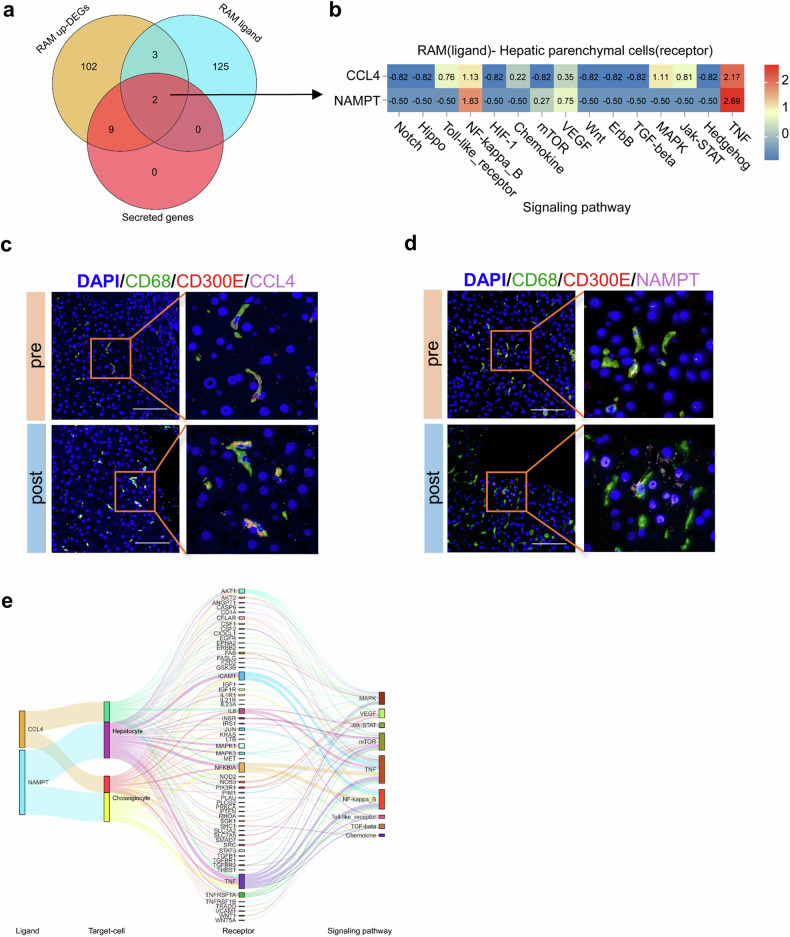
NicheNet analysis of ligand‒receptor interactions and related pathways. **a** Venn diagram showing the intersection of upregulated DEGs between groups, ligand genes, and 11 secreted genes in RAMs. **b** Heatmap showing the correlation of the two genes with signaling pathways related to liver regeneration across sets (the correlation coefficient decreases from red to blue). **c**, **d** Fluorescence mIHC image showing the coexpression of CCL4/NAMPT (pink), CD300E (red), and CD68 (green) in liver tissues before and after surgery. Scale bar, 100 μm. **e** Sankey chart showing the interactions among the 2 ligand genes, target cells, related receptors, and signaling pathways.

Following the identification of RAM subpopulations, we further analyzed cellular communication in the microenvironment, especially in CD300E^+^ RAMs. CellPhoneDB was used to predict the intercellular ligand–receptor pair relationships, and the interaction network diagram depicted the intricate interplay among cells within the hepatic immune microenvironment before and after splenectomy (Supplementary Fig. 5a). The interactions between CD300E^+^ RAMs and hepatic parenchymal cells were also altered (Supplementary Fig. 5b). The ligand‒receptor pairs whose expression significantly changed were screened, and the results were visualized (Supplementary Fig. 5c). NicheNet predicted the associations between CD300E^+^RAM and ligand‒receptor-liver regeneration-related signaling pathways in hepatic parenchymal cells. By intersecting the predicted ligands of CD300E^+^ RAMs with the above screened genes encoding secreted cytokines, CCL4 and NAMPT were identified as potential ligands that bind to the corresponding receptors on hepatic parenchymal cells. These factors may facilitate liver regeneration via the TNFα signaling pathway (Fig. 5a, b). Furthermore, fluorescence mIHC revealed that CD300E^+^RAMs in liver tissues expressed CCL4 and NAMPT before and after surgery (Fig. 5c, d). We also visualized the predicted ligand‒receptor signaling pathways (Fig. 5e). These findings provide valuable insights for future research.

### Liver regeneration after splenectomy in cirrhotic mice

**Fig. 6:**
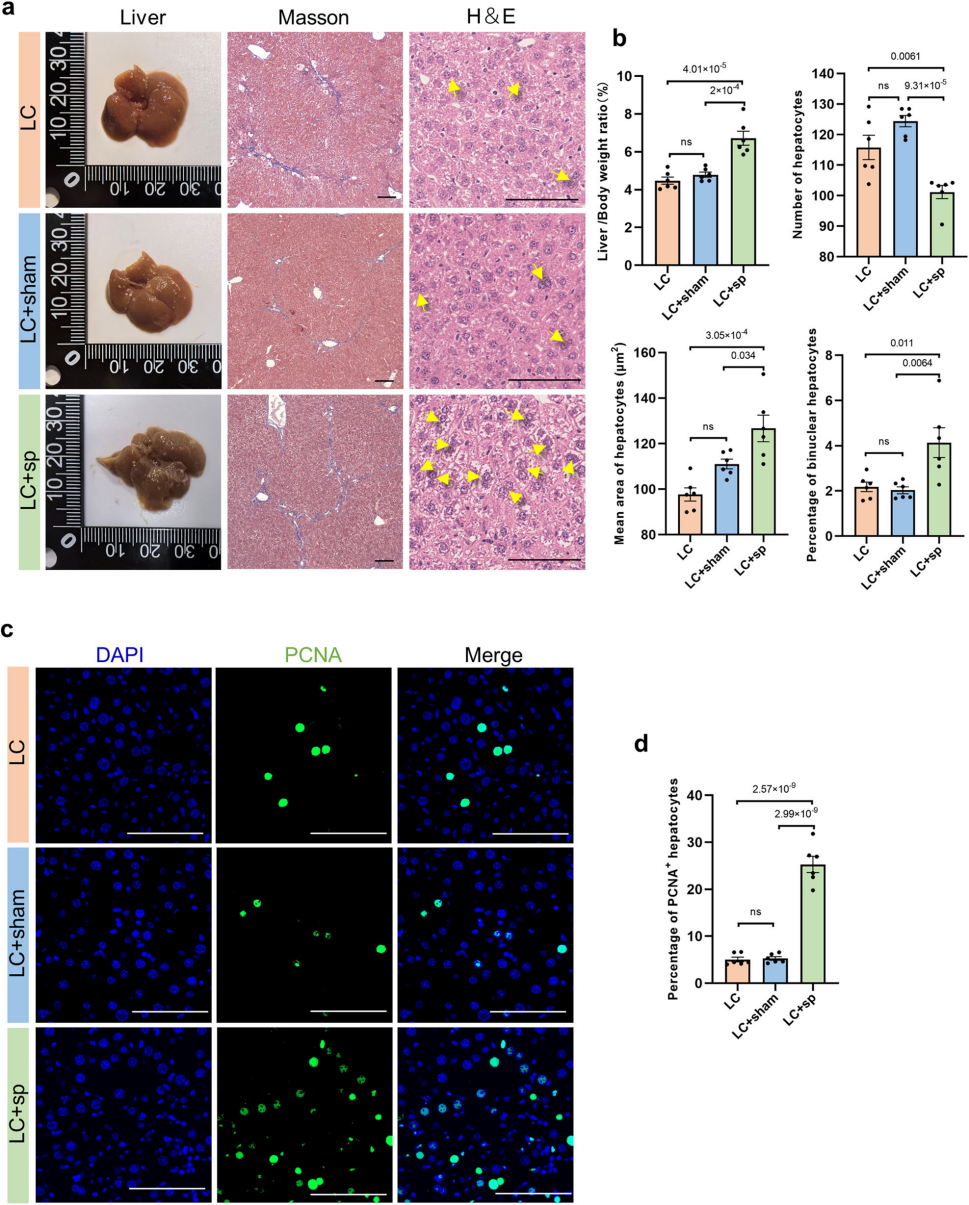
Liver regeneration after splenectomy in cirrhotic mice. **a** Gross specimen, Masson and H&E staining images of liver tissues: the yellow arrow indicates binuclear hepatocytes. *N* = 6, scale bar, 100 μm. **b** Comparison of liver index between groups, the number of hepatocytes, the proportion of binuclear cells, and the average area of hepatocytes were statistically analyzed at the same magnification and within the same area of the visual field. **c**, **d** Representative immunofluorescence images: DAPI (blue), PCNA (green), and hepatocyte proliferation were quantified by counting the PCNA-positive cells. Five randomly selected microscopic fields were captured per sample and quantified via ImageJ. The data represent the means ± SEMs, *n* = 6. Statistical significance was determined via one-way ANOVA. Scale bars, 100 μm. LC liver cirrhosis group, Sham sham-operated group, Sp splenectomy group.

The carbon tetrachloride-induced liver cirrhosis mouse model has been extensively utilized in investigations of chronic liver injury^18^. Splenectomy was performed in mice with liver cirrhosis. Two weeks postoperation, gross histology, Masson, and H&E staining (Fig. 6a) revealed a significantly higher liver index (liver weight/body weight ×%) in the splenectomy group (LC+sp) than in the cirrhosis group (LC) and sham operation groups (LC + sham). Additionally, under the same magnification and field of view, the number of hepatocytes significantly decreased in the LC + sp group. The mean hepatocyte area significantly increased, as did the proportion of binucleated hepatocytes. The LC and LC + sham groups did not significantly differ (Fig. 6b). Immunofluorescence staining revealed a significantly greater proportion of PCNA^+^ hepatocytes in the LC + sp group than in both the LC and LC + sham groups, and no significant difference was detected between the LC and LC + sham groups (Fig. 6c, d). The animal results were consistent with those of the clinical samples.

### CD300E^+^ monocyte-derived macrophages (MDMs) promote hepatocyte proliferation after splenectomy in cirrhotic mice

**Fig. 7:**
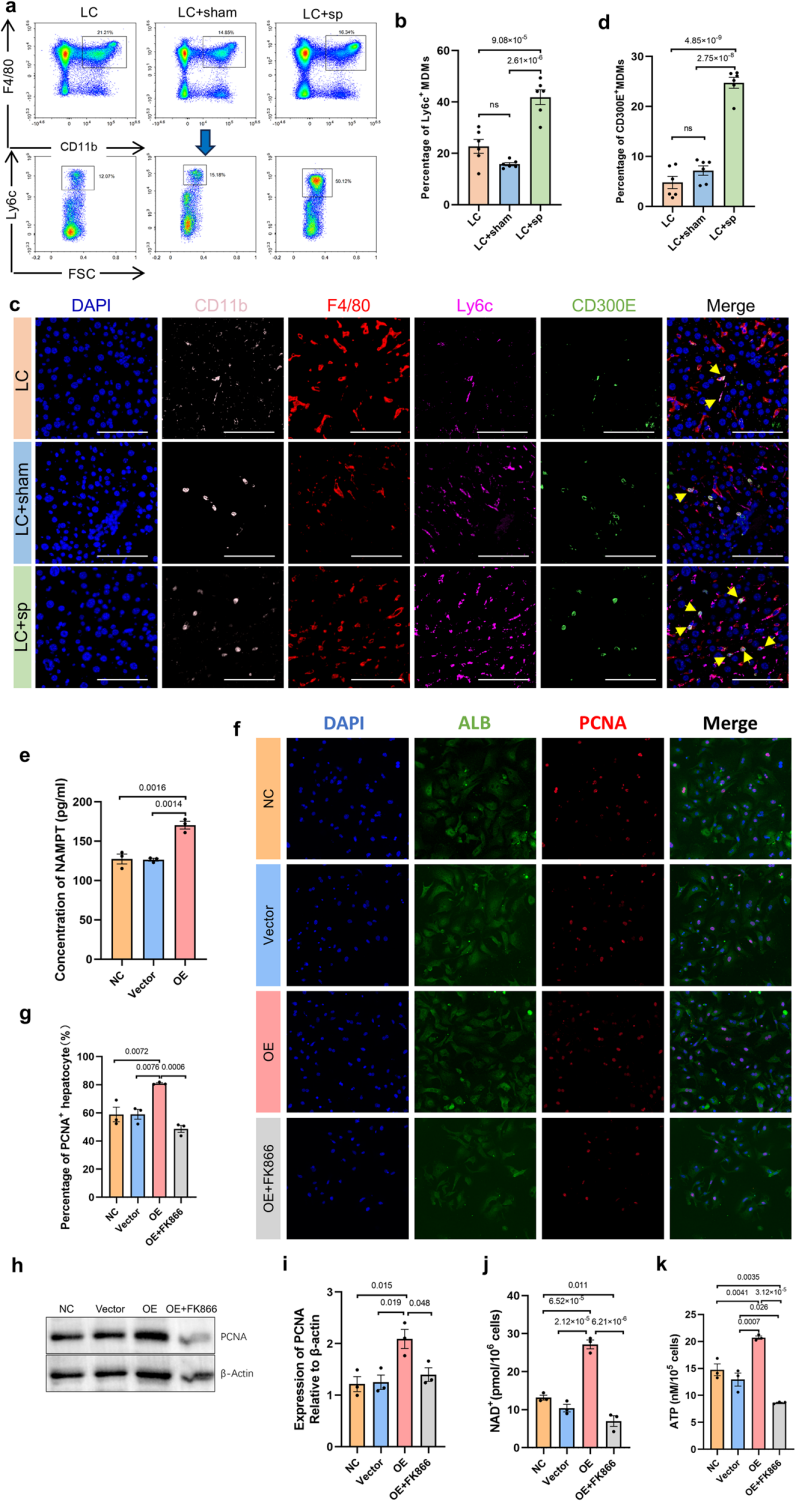
CD300E^+^ MDMs promoted hepatocyte proliferation after splenectomy in cirrhotic mice. **a**, **b** The percentage of F4/80^+^CD11b^+^Ly6c^+^ MDMs in the liver was detected via flow cytometry. **c**, **d** Fluorescence mIHC image showing the coexpression of CD11b (pink), F4/80 (red), Ly6c (rose red), CD300E (green) and DAPI (blue) in the liver tissues of the three groups. Five randomly selected microscopic fields were captured per sample and quantified via ImageJ. The data represent the means ± SEMs, *n* = 6. Statistical significance was determined via one-way ANOVA. Scale bars, 100 μm. **e** ELISA-based quantitative measurement of NAMPT concentrations in BMDM supernatants. **f**, **g** Immunofluorescence-based double staining of ALB (green) and PCNA (red) in mouse primary hepatocytes; scale bar, 100 μm. **h**, **i** Western blot analysis showing the expression of PCNA in mouse primary hepatocytes following coculture with BMDMs. **j**, **k** Intracellular NAD^+^ and ATP levels in mouse primary hepatocytes following coculture with BMDMs. *N* = 3, mean ± SEM, one-way ANOVA. NC negative control, OE overexpression. All the experiments were performed in triplicate. LC liver cirrhosis group, sham sham-operated group, sp splenectomy group, NC negative control, OE overexpression.

The results of human scRNA-seq suggested that CD300E^+^ RAMs were derived from circulating monocytes. Because CD11b and Ly6c serve as specific markers for mouse MDMs^36,37^, flow cytometry analysis of isolated liver mononuclear cells revealed a significant increase in the percentage of CD11b^+^F4/80^+^Ly6c^+^ MDMs after splenectomy in cirrhotic mice (Fig. 7a, b). Fluorescent mIHC revealed a significantly greater proportion of CD300E^+^CD11b^+^F4/80^+^Ly6c^+^ MDMs in the liver following splenectomy than in the LC and LC + sham groups (Fig. 7c, d).

To further validate the function of CD300E^+^ MDMs, primary monocytes were isolated from mouse bone marrow and then differentiated into BMDMs by adding M-CSF. The purity of the BMDMs, as determined by flow cytometry, was 96.84 ± 1.4% (Supplementary Fig. 6a). CD300E-overexpressing (OE) BMDMs were constructed via transfection of the OE-CD300E plasmid, and the transfection efficiency was validated via GFP fluorescence and quantitative reverse transcription‒PCR (qRT‒PCR) (Supplementary Fig. 6b, c). The level of NAMPT in the culture supernatant significantly increased after CD300E overexpression, as detected by ELISA (Fig. 7e). NAMPT is a rate-limiting enzyme for intracellular NAD^+^ synthesis that affects cell proliferation by regulating cellular energy metabolism^38^, and FK866 is a specific inhibitor of this process. To further investigate the effect of CD300E^+^ MDMs on hepatocyte proliferation, we established a Transwell coculture system of CD300E-OE BMDMs and primary hepatocytes, in which hepatocytes were seeded in the upper chamber and CD300E-OE BMDMs were seeded in the lower chamber. We detected an increased proportion of PCNA^+^ hepatocytes in the BMDM-overexpressing CD300E group compared with the other groups (Fig. 7f, g). Western blot analysis further confirmed a significant increase in the protein expression of PCNA in the CD300E-OE group compared with the other groups (Fig. 7h, i). Moreover, we examined the levels of NAD^+^ and its downstream ATP, which are related to cellular energy metabolism in hepatocytes after coculture, and these factors were significantly elevated in the OE group, which corresponded to the hepatocyte proliferation results (Fig. 7j, k). Therefore, CD300E^+^ MDMs regulate the energy metabolism of hepatocytes by secreting NAMPT, which may be the intrinsic mechanism of hepatocyte proliferation after splenectomy in patients with cirrhosis.

## Discussion

The hepatic immune microenvironment plays an important role in liver regeneration, which is a unique and irreplaceable process that promotes liver repair in response to diverse injurious stimuli^39^. The activation of liver regeneration leads to the restoration of liver volume via increases in hepatocyte size and cell proliferation, thereby maintaining normal organ functions^39,40^. As the largest secondary lymphoid organ in the human body, the spleen is essential for maintaining immune homeostasis. It interacts with the liver to jointly modulate the hepatic immune microenvironment^41^. Some studies have revealed that splenectomy can suppress or limit the progression of liver fibrosis^42^ and promote hepatocyte proliferation through various classical pro-proliferative signal transduction pathways^43^. Our previous study also demonstrated that splenectomy increases liver volume in patients with cirrhosis via classical liver regeneration^7^. In the present study, splenectomy not only effectively relieved hypersplenism but also improved liver function and coagulation-related indices in cirrhotic patients with hypersplenism, which was consistent with the findings of previous studies^5^. In addition, pathology results revealed enlarged hepatocytes and an increased proportion of binucleated hepatocytes after surgery. Hepatocyte proliferation was confirmed by PCNA immunofluorescence staining. These findings are consistent with those of other liver regeneration-related studies^44^.

Using scRNA-seq, we elucidated the changes in the hepatic immune microenvironment after splenectomy for the first time at the single-cell level. The results revealed significant changes in the proportions and compositions of certain immune cell subsets. The proportion of intrahepatic T cells significantly increased, whereas the proportion of mononuclear phagocytes markedly decreased. Lymphocytes represent the predominant components of the spleen, with T cells comprising approximately 40% of the population. Notably, in patients with cirrhosis complicated by hypersplenism, T-cell proportions are substantially reduced, whereas splenectomy results in a considerable increase in T-cell prevalence^45,46^. Our findings are consistent with this pattern, and the observed decrease in the proportion of mononuclear phagocytes is due primarily to the increase in T-cell percentages. Recent studies have shown that macrophages in intrahepatic nonparenchymal cells often act as effector cells that influence the course of cirrhosis and liver regeneration^39^. Because liver macrophage heterogeneity is closely related to liver regeneration^47^, we focused on the relevant macrophage subsets in subsequent experiments.

Macrophages play important roles in maintaining liver homeostasis and regeneration^48,49^. The liver serves as the primary reservoir for resident macrophages, with hepatic macrophages residing in the sinusoids accounting for 80–90% of the total macrophage population^50^. The main sources of hepatic macrophages include resident hepatic macrophages, termed Kupffer cells, and infiltrating bone marrow-derived macrophages, which originate from circulating monocytes^37^. Macrophages exhibit significant heterogeneity, perform different functions, and display diverse phenotypes within the hepatic immune microenvironment^47^. Resident macrophages in the liver are exhausted by chronic liver injury, leading to the replenishment of MDMs, which then become the dominant hepatic macrophages that affect liver regeneration^36,39,51^. In the inflammatory recruitment process, the spleen serves as a reservoir for monocytes by supplying a substantial number of monocytes. After splenectomy, the bone marrow continues to generate monocytes that directly enter the sites of inflammation^52^. Our study characterized multiple distinct subsets of intrahepatic macrophages. Among these subsets, the proportion of CD300E^+^ macrophages increased markedly after splenectomy. Based on RNA velocity and pseudotime trajectory analyses, we also observed the propensity of monocytes to differentiate into CD300E^+^ macrophages. Furthermore, monocytes express increased levels of genes involved in differentiation, such as genes related to oxidative phosphorylation^53^, protein modification^54^, and cell metabolism^55^. This observation aligns with the current understanding of the multifunctionality, heterogeneity, and plasticity of macrophages. In addition, this finding is consistent with the fact macrophages can be induced to exhibit specific functional phenotypes in response to particular microenvironmental stimuli or cytokines^34^.

We found that CCL4 and NAMPT are secretory genes that are highly expressed in CD300E^+^ macrophages after splenectomy. Previous studies have demonstrated that CCL4 plays a pivotal role as a chemokine in orchestrating both acute and chronic inflammatory responses at sites of injury or infection, mainly by recruiting proinflammatory cells and facilitating the migration of T cells from the circulation to inflamed tissues by binding to the cell surface C-C chemokine receptor 5 (CCR5)^56^. This finding may explain the observed increase in CD300E^+^ macrophages after splenectomy, which recruit T cells through CCL4 secretion to significantly increase the proportion of T cells. Moreover, as a rate-limiting enzyme in the biosynthesis of NAD^+^, NMAPT affects cellular energy metabolism and participates in cell differentiation and proliferation, and its cytokine-like function has attracted considerable attention in recent years^38^. CD300E^+^ macrophages may affect hepatocyte energy metabolism and promote hepatocyte proliferation by secreting NAMPT. Additionally, our analysis of cell‒cell interactions via NicheNet indicated that NAMPT may synergize with the TNF signaling pathway to promote hepatocyte proliferation and regeneration. Consistent with existing research, TNF signaling is closely associated with the initiation of liver regeneration and influences hepatocyte proliferation by activating multiple downstream signaling pathways^40^. NAMPT may modulate the cellular response to TNF signaling by regulating energy metabolism, potentially providing a new avenue for exploring the mechanisms of liver regeneration. Based on the results of the intercellular interaction analysis, NAMPT may also play a role in promoting liver regeneration via cytokine-like functions by binding to the corresponding receptors in liver parenchymal cells.

Furthermore, we observed liver regeneration in a mouse model of cirrhosis with splenectomy, similar to that observed in human samples. To further investigate the mechanism by which CD300E^+^ macrophages promote liver regeneration, we cocultured mouse primary BMDMs with hepatocytes. The results showed that CD300E^+^ macrophages may contribute to the promotion of hepatocyte proliferation by facilitating NAD^+^ synthesis via the activation of NAMPT. Previous studies have demonstrated that the overexpression of NAMPT promotes liver regeneration in mice after partial hepatectomy by affecting NAD^+^ synthesis^57^. NAD^+^ is an essential metabolite for energy metabolism, which is a key cellular process that includes ATP generation, glycolysis, mitochondrial respiration, and other metabolic reactions within the cell^57,58^. We observed that ATP content, which is associated with cellular energy metabolism, was significantly increased in hepatocytes in the overexpression culture system and significantly decreased after FK866 treatment. Combined with the results of PCNA cellular immunofluorescence, we confirmed that CD300E^+^ BMDMs promote hepatocyte proliferation by increasing NAMPT secretion, thereby increasing intracellular NAD^+^ and ATP synthesis related to cellular energy metabolism in hepatocytes.

In this study, we hypothesized that splenectomy promotes liver regeneration by regulating the cell composition of the immune microenvironment. Using scRNA-seq, we investigated this phenomenon and generated a comprehensive immune microenvironment map of liver cirrhosis after splenectomy. We characterized macrophage subsets within the hepatic immune microenvironment, elucidated the correlation between CD300E^+^ macrophage subsets and liver regeneration, and validated the mechanism by which CD300E^+^ macrophages influence hepatocyte energy metabolism to facilitate liver regeneration via NAMPT secretion. This study describes the single-cell landscape of the hepatic immune microenvironment to facilitate further exploration of the mechanisms underlying liver regeneration and liver–spleen crosstalk in cirrhosis. Our findings also highlight the potential of CD300E^+^ macrophages as novel therapeutic targets for patients with chronic liver injury.

## Supplementary information

Supplementary Information

## Data Availability

The scRNA-Seq data generated in this study were deposited in the Genome Sequence Archive (GSA) for the Human database under the accession code HRA005061 (https://ngdc.cncb.ac.cn/gsa-human/).
