# Supplementary material for: CD300E+ macrophages facilitate liver regeneration after splenectomy in decompensated cirrhotic patients
Source: Exp Mol Med. 2025 Jan 1;57(1):72–85. doi: 10.1038/s12276-024-01371-3 (PMC11799435; doi:10.1038/s12276-024-01371-3)
Supplement: Supplementary file 1 — Supplementary Information [file 12276_2024_1371_MOESM1_ESM.pdf]

CD300E$^+$ macrophages facilitate liver regeneration after splenectomy in decompensated cirrhotic patients

Tao Yang[1], Yuan Zhang[2], Chujun Duan[2], Hui Liu[1], Dong Wang[1], Qingshan Liang[1], Xiao Chen[1], Jingchang Ma[2], Kun Cheng[2], Yong Chen[3], Ran Zhuang[2,*], Jikai Yin[1,*]

Contents

Supplementary materials and method 3

Supplementary Figures and Legends 14

Supplementary Tables 22

Abbreviations 26

Supplementary materials and method

Patients

Inclusion criteria: 1) hepatitis B-related cirrhosis; 2) portal hypertension with varicose bleeding in cirrhosis; 3) no history of variceal bleeding but at high risk of variceal bleeding (cherry red spots for grade III esophagogastric variceal, blue variceal, or hemorrhagic variceal veins on gastroscopy, combined with severe hypersplenism (WBC < 2.0×10^9/L, PLT < 50×10^9/L[1, 2], HVPG > 12 mmHg); 4) pathological diagnosis of splenomegaly with stasis. Exclusion criteria: 1) liver function as Child-pugh grade C; 2) over 70 years of age or complication with liver cancer.

H&E staining

Paraffin sections were dewaxed by two incubations with xylene (20 min each), followed by treatment with anhydrous ethanol (5 min, twice) and 75% alcohol (5 min). After washing with distilled water, hematoxylin solution was added for 3-5 min. This was followed by washing with water and incubation with eosin (5 min). Dehydration was performed with 85% and 95% gradient alcohol (5 min each). Next, anhydrous ethanol was used to treat the samples thrice (5 min each). Transparent neutral gum sealing was performed before microscopy for image collection and analysis.

Masson’s trichrome stain

Paraffin sections were dewaxed by two incubations with xylene (20 min each), followed by treatment with anhydrous ethanol (5 min, twice) and 75% alcohol (5 min). After

washing with distilled water, incubation with Masson A liquid was performed overnight, followed by washing in distilled water. Next, Masson B and Masson C solutions were successively added for 1 min each. After washing, differentiation was performed with 1% hydrochloric acid alcohol. After successive incubations with Masson D (6 min), Masson E (1 min) and Masson F (2-30 s) solutions, the slices were rinsed and differentiated with 1% glacial acetic acid. Next, anhydrous ethanol was used to treat the samples thrice (5 min each). Transparent neutral gum sealing was performed before microscopy for image collection and analysis.

Fluorescent multiplex immunohistochemistry (mIHC)

The sections were washed successively with environmental dewaxing solutions I, II and III (10 min each), followed by treatment with anhydrous ethanol (5 min, twice) and 75% alcohol (5 min). Antigen repair was performed with EDTA (pH9.0) in a microwave with medium heat for 8 min and medium-low heat for 7 min. After cooling at ambient, slides were washed with PBS (PH7.4) with shaking on a decolorizing shaker thrice (5 min each time). After treatment with 3% hydrogen peroxide at room temperature shielded from light for 25 min, blocking with 3% BSA was performed for 30 min. This was followed by overnight incubation with primary antibodies in a wet box at 4°C. Then, the corresponding HRP-labeled secondary antibodies were added for 50 min at room temperature. After three washes with PBS (PH7.4), TSA was added for incubation at room temperature in the dark for 10 min. Next, samples were washed with TBST 3 times on a shaker for 5 min each. Antigen retrieval was performed as described above.

After blocking, the corresponding primary and secondary antibodies were added successively, followed by incubation with the TSA dye. DAPI counterstaining was performed at room temperature away from light for 10 min. For self-fluorescence quenching, samples were washed with PBS (PH7.4) on a shaker for 3 times (5 min each), followed by addition of an autofluorescence quencher (5 min) and water rinsing for 10 min. Next, anti-fluorescence quenching sealing tablets were used for mounting.

Preparation of single-cell suspensions from tissue samples

Liver tissue samples were placed in C Tubes with gentleMACS™ Octo Dissociator, and dissociation was performed for approximately 30 min. Each suspension was filtered through a 70-μm filter attached to a 50 ml centrifuge tube. Digestion was terminated with 8 ml of the digestive termination solution. The samples were transferred to 15 ml centrifuge tubes and centrifuged at 400g for 5 min, followed by addition of 1.5 ml of cell lysis solution and incubation on ice for 5 min. Precooled 5 ml PBS-resuspended cells were centrifuged at 4℃ and 400g for 5 min. The supernatant was discarded, and the process was repeated twice. The cells were re-suspended with 1 ml of precooled PBS, and the cell suspension was filtered with a 40-μm screen. The resulting cell suspension was centrifuged at 400g for 5 min, and cells were finally resuspended in 500μl PBS.

Cell quality inspection and sequencing library construction

Each single-cell suspension was mixed with 0.4% Trypan Blue dye at a 9:1 ratio. Cells were counted with Countess® II Automated Cell Counter and the proportion of live cells was determined. The quality control standard was more than 90% viability, with cell density not below 1000 cells/L. Single cells were isolated with 10x Genomics apparatus (3'v3) to form GEMs (Gel Beads In-Emulsions). GEMs flowed into a reservoir and were collected. The gel beads dissolved and released Barcode sequences, which were reverse transcribed and labeled. The gel beads and oil droplets were digested, and PCR amplification was performed with the cDNA as template. Standard sequencing library was constructed by mixing all GEMs products. The cDNA libraries were sequenced on an Illumina sequencing platform by Genedenovo Biotechnology Co., Ltd (Guangzhou, China).

The paired-end sequencing mode of the Illumina sequencing platform was used for high-throughput sequencing of the constructed library. At Read1 end, the information about 16 bp barcodes and 10 bp UMIs (unique molecular identifiers) was used to quantify cell numbers and expression levels. At Read2 end, the cDNA fragment served as a reference for genome alignment to determine the gene corresponding to the mRNA.

Data quality control and cell cluster analysis

Cell Ranger (version 5.0.0) was used for quality control of the original data, and reads were compared to the reference genome using the STAR (Spliced Transcripts Alignment to a Reference) comparison software. barcodes and UMIs were further

filtered and corrected to generate the original gene expression matrix. The cell-by-gene matrices for each sample were individually imported to Seurat (version 3.1.1) [3]for downstream analysis. High-quality cells were further selected for retention, with the following criteria: a. DoubletFinder (version 2.0.3) [4] was used to determine the multicellular rate of each sample for multicellular filtering; b. the number of genes identified in a single cell was 200-5000, and a barcode with higher or lower gene number was eliminated; c. the total number of UMIs in a single cell was limited to 0-20000, and cells with a higher UMI total number were eliminated; d. the proportion of mitochondrial gene expression in single cells was set at 0-50%, and cells with higher mitochondrial gene expression were excluded. Gene expression levels were homogenized, and Harmony [5] was used for data consolidation and batch effect correction. The cells were grouped by principal component analysis (PCA) [6].

Clusters visualization

High-dimensional cell data were mapped into the two-dimensional space with t-distributed Neighbor Embedding (tSNE) [7]. Cells with similar expression patterns were grouped and those with different expression patterns were separated to more intuitively show differences between cells. In order to further confirm the cell clusters identified by tSNE, uniform manifold approximation and projection (UMAP) analysis was performed [8]. Assuming that the data samples were uniformly distributed in manifold topological spaces, approximation and projection could be made from the data samples to low-dimensional spaces.

Cell type annotation

Cell annotation was performed with singleR (version 1.0.5) [9]. Pearson correlation coefficients were calculated for all cell types in the target cell and all samples in the reference data set, and a similarity value for each cell corresponding to each cell type was obtained by integrating the data. Cell types were automatically identified by the similarity between cells and cell types in the reference database. Annotation accuracy for highly similar cell types would be reduced, so we combined manual annotation to finally confirm the identity of the cell subsets.

Differentially expressed (upregulated) gene analysis

The expression level of each gene in a given cluster was compared against the remaining cells by the Wilcoxon rank sum test [10]. Significantly upregulated genes were identified based on the following criteria: a. $|\log2FC| > 0.5$； b. $p < 0.05$； c. gene expressed in at least 25% of the cells in a subpopulation.

Enrichment analysis

Gene ontology (GO) [11] is an internationally standardized classification system for gene functions, which could comprehensively describe the properties of genes and gene products in organisms. Each GO term corresponds to an attribute. Differentially expressed genes were mapped to each term of the GO database

(http://www.geneontology.org/), and the number of genes in each term was calculated. The hypergeometric test was applied to analyze the Biological Processes (BPs) of DEGs by GO functional enrichment, and significantly enriched GO entries were screened ($p < 0.05$). Kyoto encyclopedia of genes and genomes (KEGG) [12] is a major public database on Pathway genomes. Pathway enrichment analysis using KEGG Pathway as a unit, the hypergeometric test was applied to identify pathways with significant enrichment ($p < 0.05$). Significant enrichment of a pathway can identify the major signal transduction pathways involving the DEGs.

RNA Velocity and pseudo-time analysis

Velocyto (velocyto.R_0.6) [13] was used to perform RNA rate analysis with the BAM file output from Cell Ranger, and arrows were used for insertion into the UMAP obtained by Seurat dimension reduction to show the differentiation trend of cells. Monocle（version 2.10.1) [14] was used to analyze cell gene expression, construct single-cell loci, reduce dimensions and visualize loci. The occurrence of branch nodes in a single cell locus represented the procedural changes in cells. During development, when cells underwent differentiation, branches appeared on the locus. Monocle used branched expression analysis modeling (BEAM) [15] to analyze the quasi-time-sorted cell data and designated nodes, and found cladding-dependent gene expression by expressing the problem as a comparison between two negative binomial GLMs.

Cell interaction analysis

CellphoneDB (version 2.0)[16] was used to analyze the expression abundance of ligand-receptor interactions between two cells. Cells in which receptors and ligands were expressed in > 10% of a given cluster were analyzed. All cell types in the dataset were compared in pairs using cellphoneDB and the number of significantly enriched ligand-receptor interactions between the two cell types was determined. We randomly arranged cluster labels for all cells (1000 times by default) and determined the average ligand expression levels in a cluster as well as the average receptor expression levels in the interacting cluster. Thus, we generated a zero distribution for each pair of ligand-receptors in each pair of comparisons between the two cell types. We obtained a *p*-value for the likelihood of cell type enrichment for each ligand-receptor complex by assessing the proportion of the mean that is equal or higher than the actual mean. $p < 0.05$ indicated significant enrichment. Based on the analysis of the number of significantly enriched ligand-receptor pairs between cell types, the cell interaction network map was constructed, which could more intuitively display the regulatory relationship between cells.

Ligand activity analysis was performed with NicheNet （Nichenet.R_1.0.0)[17] to prioritize the gene expression of sender cell ligands most likely to influence the interaction with recipient cells. This process is termed ligand activity prediction, and the ligands were sequenced according to the correspondence between their previous target gene predictions and the changes in gene expression observed in the sending cell

communication. Using NicheNet, which contains protein-protein interactions from ligands to downstream signaling pathways, as well as the interactions between signaling pathways and target genes, regulatory potential scores between ligands and target gene pairs were determined.

Immunofluorescence

Paraffin sections were dewaxed for rehydration and repaired with an antigen repair solution. Blocking was conducted in 5% bovine serum albumin (BSA) (Boster Biological Technology, AR0004) for 30min. Primary antibody PCNA (Proteintech Group, 10205-2-AP) was added and incubated overnight at 4 ° C. Cy3-conjugated goat anti-rabbit secondary antibody (Proteintech Group, SA00009-2) was incubated for 1 h at room temperature. The sections were stained and sealed with an anti-fluorescence quenching sealant containing DAPI (Servicebio G1407-25ml), then photoed by fluorescence microscopy. For cellular immunofluorescence, cell climbing slices were washed with PBS, fixed with 4% paraformaldehyde, and then incubated with 0.5% triton X-100 for permeabilization. Blocking was conducted in 5% BSA for 30min. Primary antibody PCNA and ALB (Proteintech Group, 16475-1-AP） were added and incubated overnight at 4 ° C. Cy3-conjugated goat anti-rabbit and ColaLite488-conjugated goat anti-mouse (Proteintech Group, CL488-25671) secondary antibodies were incubated for 1 h at room temperature. Slides were washed and incubated for 8 min with DAPI (Servicebio G1012-100ml), sealed with an anti-fluorescence quenching sealant and photoed by fluorescence microscopy.

Isolation of mouse bone marrow monocytes

Monocyte isolation was performed according to the manufacturer's instructions of mouse bone marrow monocyte isolation kit (TBD, TBD2013DM). 6–8 weeks old C57BL/6 mice were sacrificed by cervical dislocation after anesthesia, femurs and tibias were collected, bone marrow tissues were flushed out of the bone marrow cavity with homogenate rinse fluid (TBD, F2013TBD). Cell suspensions were filtered through the 70μm cell strainer (Corning Inc., Corning, NY) after repeatedly blowing, and centrifuged at 450 g for 10 minutes, the supernatant was discarded, cells were resuspended in sample diluent (TBD, 2010C1119). Cell suspension was slowly added to the gradient separation solution, and centrifuged at 450 g for 30 minutes. The upper cloud layer was collected and washed with wash solution (TBD, 2010X1118), centrifuged at 400g for 10min, and the supernatant was discarded.

Plasmid Construction

For overexpression of CD300E, we used the *pEGFP-N1* construct containing *Cd300e* full length cDNA，Transcripts is： Mus musculus -NM_172050.3.

qRT-PCR tests

The total RNA of cell samples was isolated using TRIGene Reagent (GenStar, P118-05). The reverse transcription was conducted using Hifair III 1st Strand cDNA

Synthesis SuperMix kit (Yeasen, 11141ES60) according to the manufacturer's instructions. The gene expression was quantitatively analyzed by Hieff® qPCR SYBR Green Master Mix (Yeasen, 11201ES08) using AriaMx Real-Time PCR System (Agilent Technologies). The total primers and probes for detecting *Cd300e* and β-actin were designed based on the published gene sequences (NCBI). The expression level for the gene was normalized to β-actin.

Antibodies

**Name**	**Supplier**	**Cat no.**
anti-CD68	Cell Signaling Technology	76437
anti-CD300E	Invitrogen	PA5-53324
anti-CCL4	Invitrogen	710391
anti-NAMPT	Invitrogen	PA5-34858
Anti-CD11b	Servicebio	GB11058
anti-F4/80	Servicebio	GB113373
anti-Ly6c	Novus	NBP3-11242
anti-CD300E	Stjohnslabs	STJ98749
DAPI	Servicebio	G1012-100ML
PCNA Monoclonal antibody	Proteintech Group	10205-2-AP
Cy3–conjugated Affinipure Goat Anti-Rabbit IgG(H+L)	Proteintech Group	SA00009-2
DAPI	Servicebio	G1407-25ml
Albumin Polyclonal antibody	Proteintech Group	16475-1-AP
CoraLite Plus 488-conjugated CHCHD10 Polyclonal antibody	Proteintech Group	CL488-25671
FITC-anti-CD45	Biolegend	103108
PE-anti-CD11b	Biolegend	101208
PE-Cy7-anti-F4/80	Biolegend	123114
Alexa Fluor 700-anti-Ly6c	Biolegend	128024
anti-β actin	Servicebio	ZB15001-HRP-100

Supplementary Figures and Legends

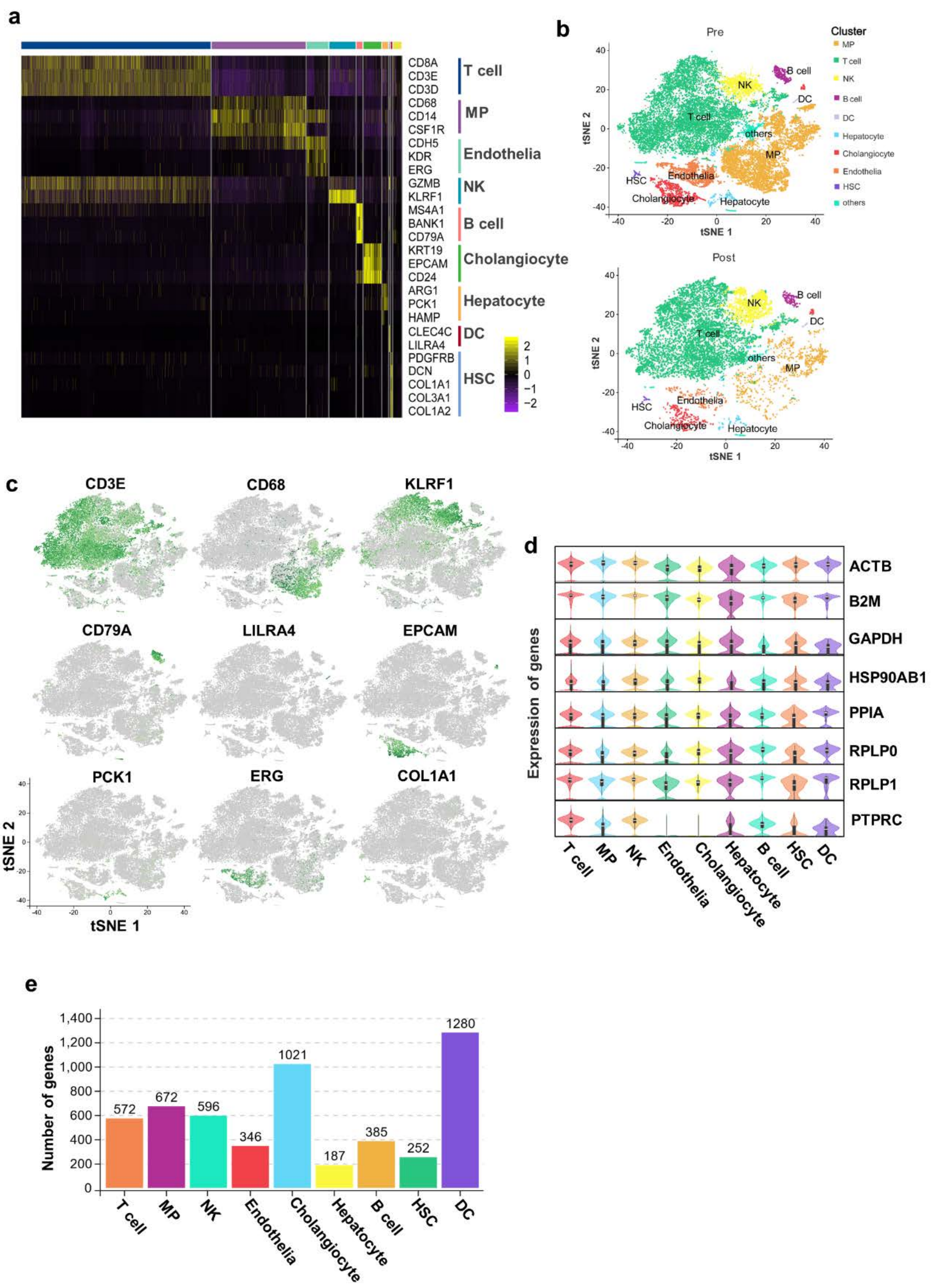

Supplementary Fig. 1 Marker genes and upregulated DEGs in each cell subset.

a Heat map showing the marker genes for cell subset annotation (expression decreases from yellow to purple). **b** t-SNE presents the results of preoperative (top) and postoperative (bottom) cell subset annotation. **c** Distribution of characteristic marker gene t-SNE in each cell subset, with green color indicating high gene expression. **d** Violin plot shows the expression of housekeeper genes in each cell subpopulation. **e** Bar chart shows the number of upregulated genes in each cell subset.

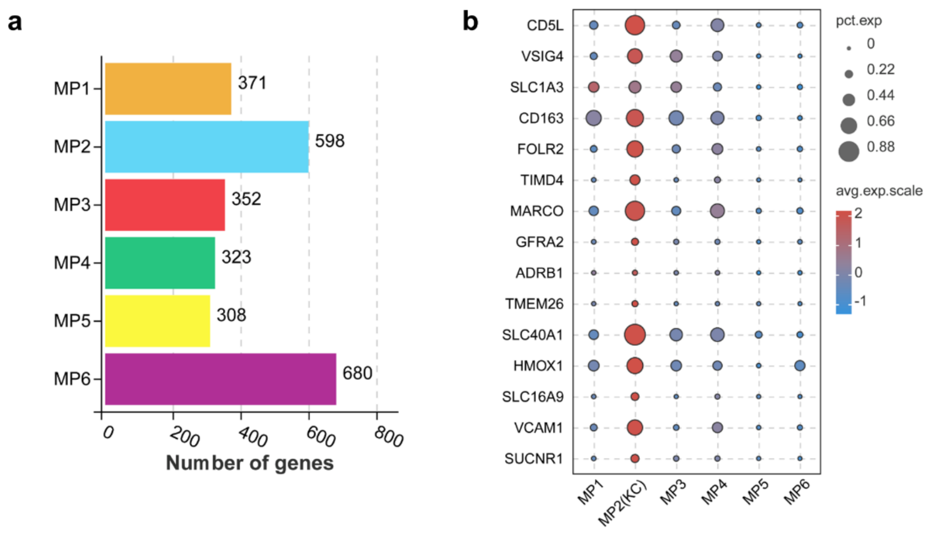

Supplementary Fig. 2 Upregulated gene expression in Mononuclear phagocyte (MP) subset.

a Bar chart shows the number of upregulated genes corresponding to each subpopulation. **b** Bubble chart shows the expression of known KC marker genes in different MP subpopulations.

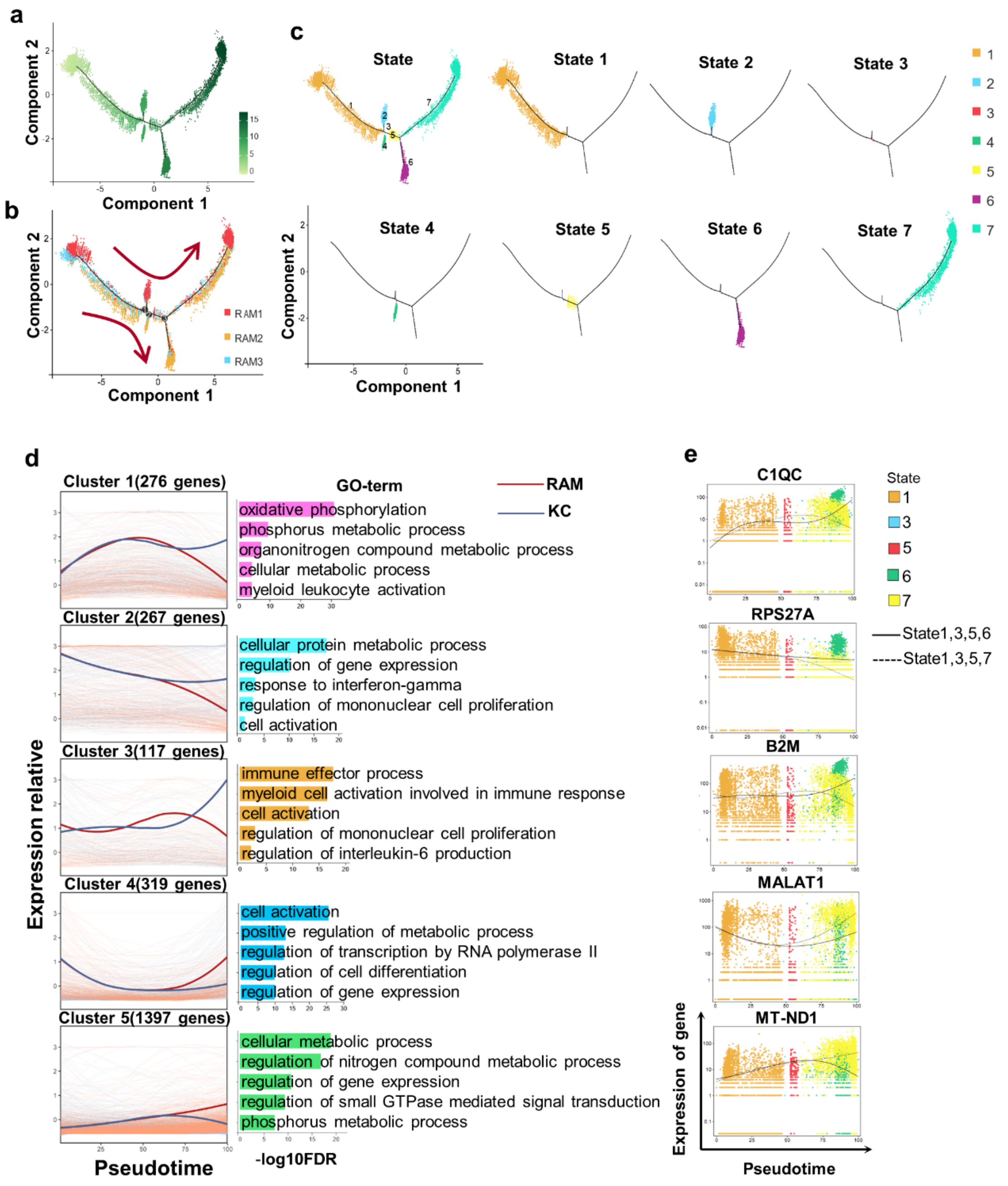

Supplementary Fig. 3 Pseudotime trajectory analysis of MP1-3 subsets.

a Cell trajectory chart shows the pseudotemporal differential status of MP1–3 cells. Each point represents a cell, and the pseudotemporal value of the legend represents the developmental stage. The smaller the pseudotemporal value is, the earlier the developmental stage is. **b** Cell trajectory chart shows the quasi-temporal development status of subsets, and different colors represent different subsets. **c** Cell trajectory chart

shows the pseudotemporal differentiation states; different branches represent different differentiation states. **d** The expression trend diagram of each cluster of DEGs (at the pseudotime branching node 3) shows differential states 1, 3, 5, and 7 in red and states 1, 3, 5, and 6 in blue. The right side shows the enriched GO terms corresponding to each cluster, and the horizontal coordinate is the value of -log10 false discovery rate (FDR) of each term. **e** Scatter plot shows the variation in the expression level of branch differential genes along the pseudotime line. The x-axis is the pseudotime axis, and the y-axis shows the expression level of the branch DEGs. Different colors represent different states, with the solid line representing States 1, 3, 5, and 6 and the dashed line representing States 1, 3, 5, and 7.

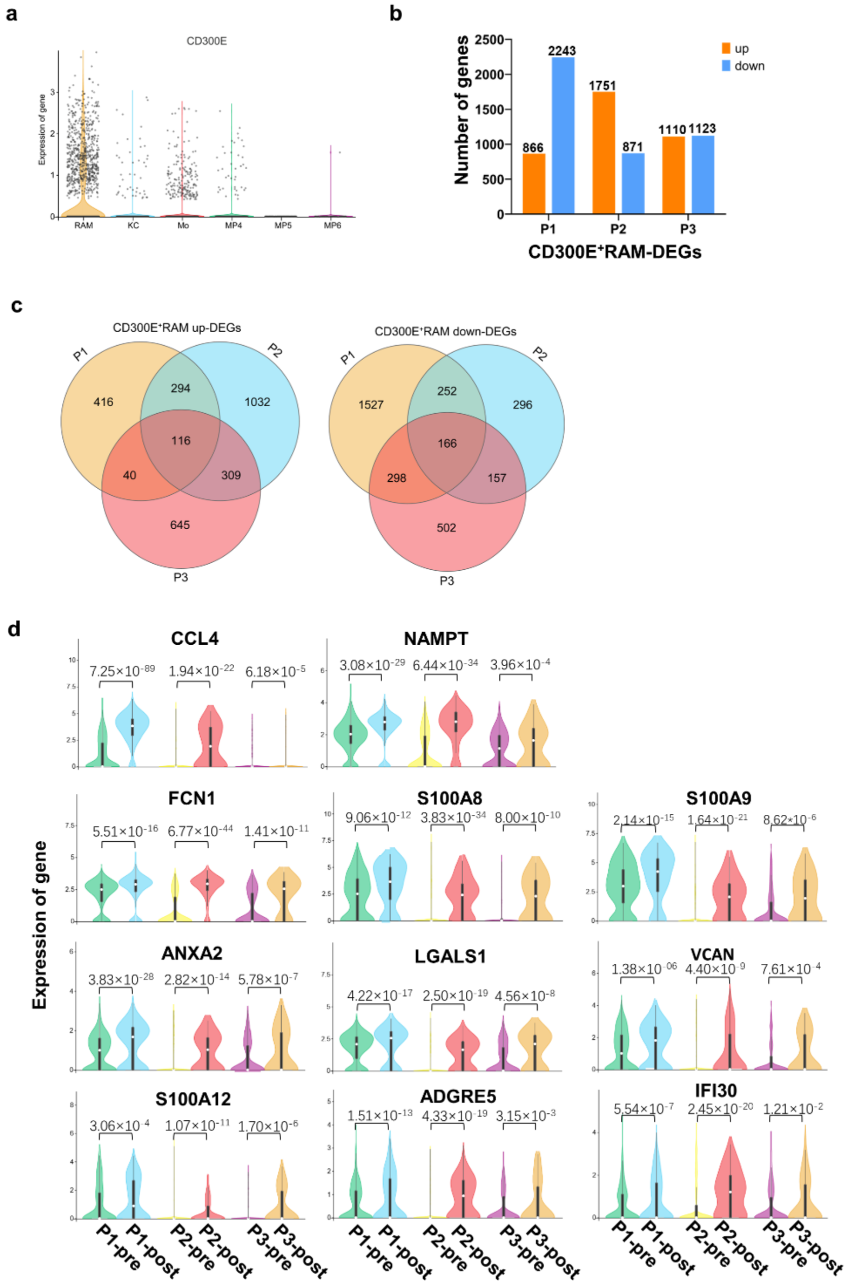

Supplementary Fig. 4 CD300E⁺ RAMs characteristic intergroup DEGs.

a Violin plot shows the expression of RAM marker CD300E in each MP subset. **b** Bar chart shows the number of DEGs in CD300E⁺RAMs between pre- and post-operation in each sample group. **c** Venn diagram illustrates the overlapping DEGs of CD300E⁺RAMs in all three groups. **d** Violin plot shows the expression of 11 differential genes in each sample group.

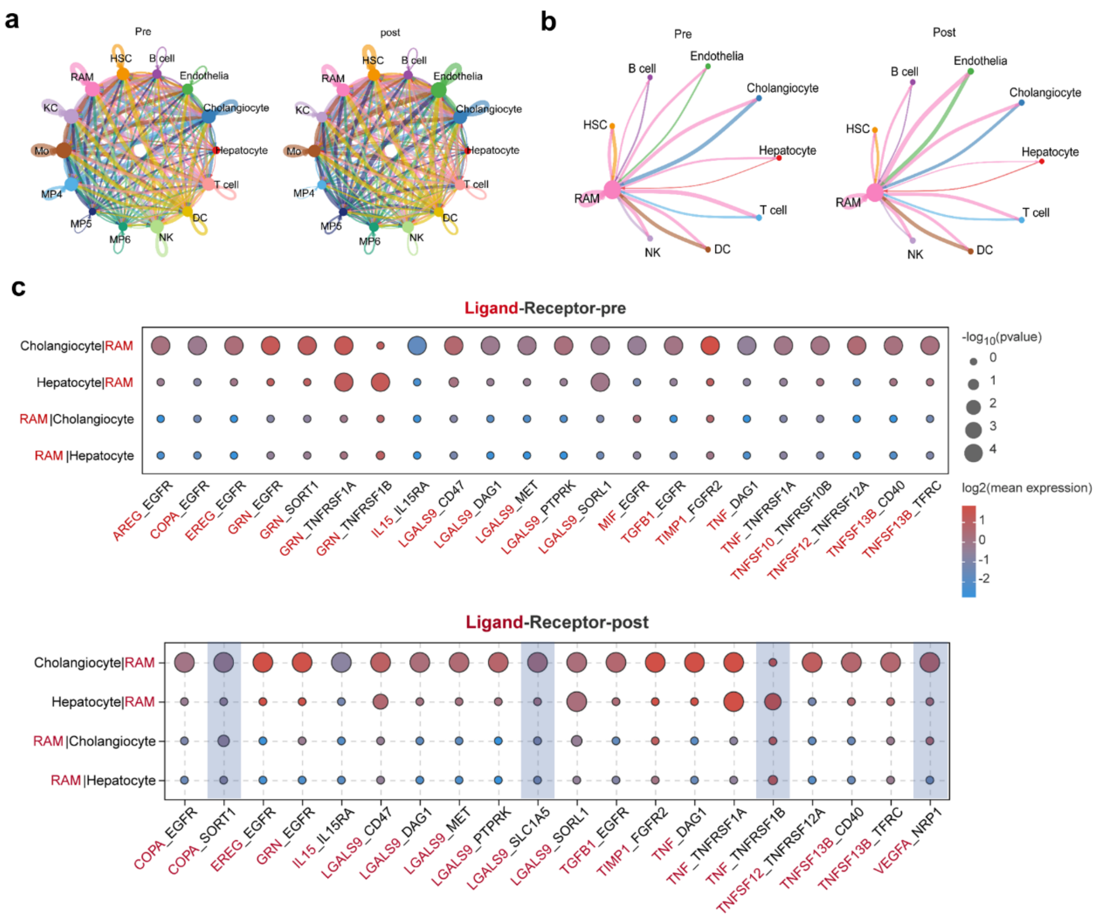

Supplementary Fig. 5 Communication between RAM cells and hepatic parenchymal cells after surgery.

a The cell interaction network chart shows the cell relationships before and after surgery, and the thickness of the lines represents the number of receptor pairs. **b** Network chart of RAM cell interaction with other cells before and after surgery. **c** Bubble chart shows the information on ligand–receptor pairs between RAM and target cells before and after surgery. The bubble color from red to blue represents the gradual decrease in the average expression of ligand–receptor pairs between cells, the bubble size from small to large represents the gradual decrease in the p value of ligand–receptor pairs, and the deepened color in the post-operation bubble chart represents the newly increased ligand–receptor pairs.

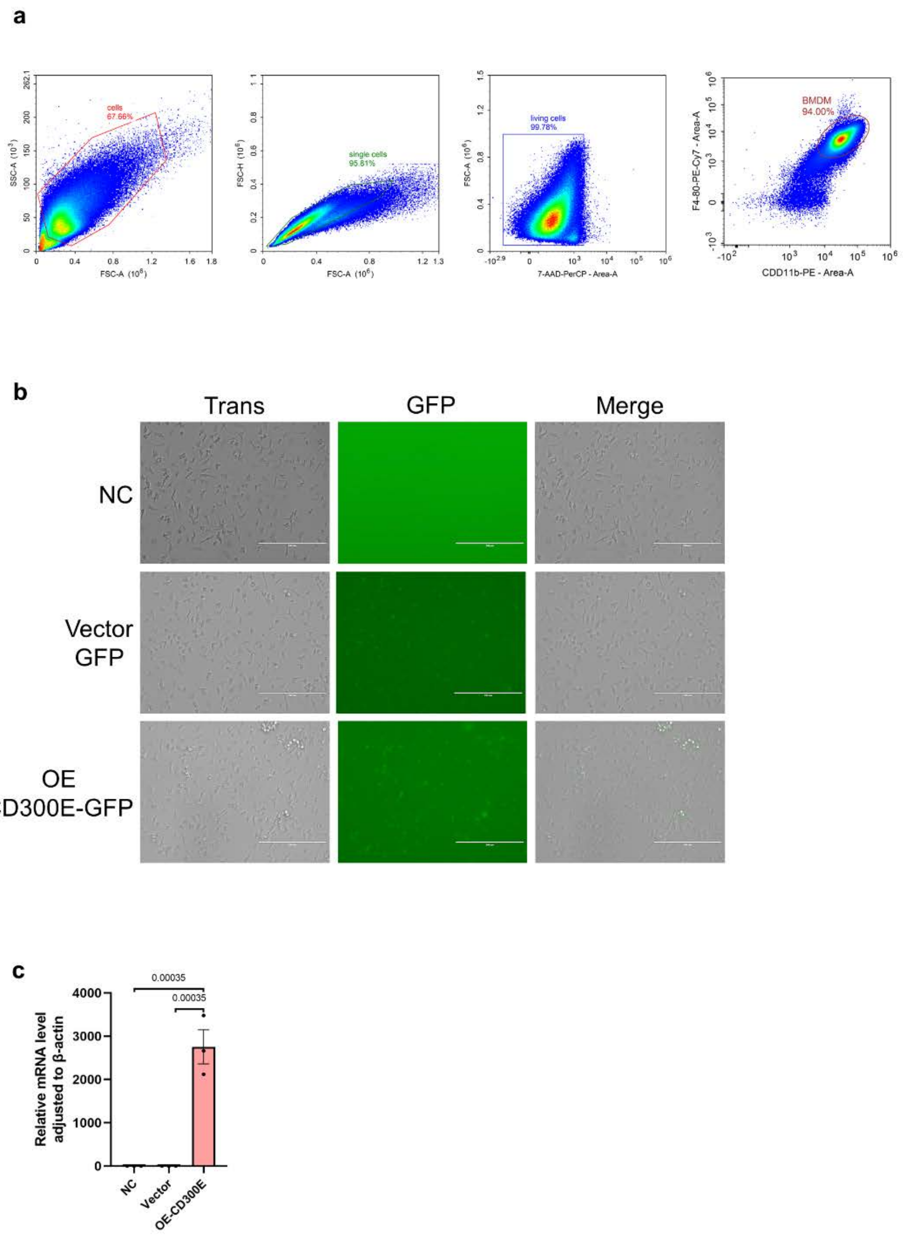

Supplementary Fig. 6 Plasmid transfection efficiency of BMDMs.

a Purity of $CD11b^{+}F4/80^{+}$BMDM was examined by flow cytometry. **b** Fluorescent images: BMDMs transfected with GFP-encoding plasmid, scale bar, 200 μm. **c** Expression of *Cd300e* in each group. N=3, mean±SEM, one-way ANOVA. All experiments were performed in triplicates. NC, negative control. OE, overexpression.

Supplementary Tables

Supplementary Table 1. Basic characteristics of preoperative individuals with liver cirrhosis included in the study.

Parameters	Values (n=54)
**Age (years)**	45.85±9.83 (26-72)
**Sex**	
Male	34
Female	20
**BMI (kg/m^2)**	22±2.68 (16.77-28.37)
**Variceal hemorrhage history**	
Positive	41
Negative	13
**ICG-R15 (%)**	17.52±13.08 (0.1-54.1)

Notes: BMI, body mass index; ICG-R15, indocyanine green retention test after 15 min.

Supplementary Table 2. Characteristics of individuals undergoing single-cell sequencing analysis.

Parameters	Patient 1		Patient 2		Patient 3	
	Pre	Post	Pre	Post	Pre	Post
**Age**	34		46		49	
**Gender**	Male		Female		Male	
**Etiology of liver disease**	HBV		HBV		HBV	
**History of EGVB**	Yes		Yes		Yes	
**WBC(×10^9/L)**	1.96	8.64	1.10	6.67	1.83	3.41
**PLT(×10^9/L)**	24	450	34	388	46	217
**RBC(×10^{12}/L)**	3.61	3.98	2.75	4.57	3.76	3.61
**TBIL(μmol/L)**	20.04	7.84	17.65	6.17	10.7	15.85
**PT(sec)**	16.4	11.3	14	12.3	13.1	12.1
**Child-pugh score**	A6	A6	A5	A5	A5	A5
**Liver volume(cm^3)**	691	835	795	1018	810	1107

Notes: Pre, pre-operation; Post, post-operation; EGVB, esophagogastric variceal bleeding.

References

1 Boyer, T. D. & Habib, S. Big spleens and hypersplenism: fix it or forget it? *Liver Int.* **35**, 1492-1498(2015).

2 Liangpunsakul, S., Ulmer, B. J. & Chalasani, N. Predictors and Implications of Severe Hypersplenism in Patients with Cirrhosis. *The American Journal of the Medical Sciences*. **326**, 111-116(2003).

3 Butler, A., Hoffman, P., Smibert, P., Papalexi, E. & Satija, R. Integrating single-cell transcriptomic data across different conditions, technologies, and species. *Nat. Biotechnol.* **36**, 411-420(2018).

4 McGinnis, C. S., Murrow, L. M. & Gartner, Z. J. DoubletFinder: Doublet Detection in Single-Cell RNA Sequencing Data Using Artificial Nearest Neighbors. *Cell Syst.* **8**, 329-337(2019).

5 Korsunsky, I. et al. Fast, sensitive and accurate integration of single-cell data with Harmony. *Nat. Methods*. **16**, 1289-1296(2019).

6 Chung, N. C. & Storey, J. D. Statistical significance of variables driving systematic variation in high-dimensional data. *Bioinformatics*. **31**, 545-554(2015).

7 Laurens Van Der Maaten, G. H. Visualizing Data using t-SNE. *Journal of Machine Learning Research*, 2579-2605(2008).

8 Becht, E. et al. Dimensionality reduction for visualizing single-cell data using UMAP. *Nat. Biotechnol.*(2018).

9 Aran, D. et al. Reference-based analysis of lung single-cell sequencing reveals a transitional profibrotic macrophage. *Nat. Immunol.* **20**, 163-172(2019).

10 Camp, J. G. et al. Multilineage communication regulates human liver bud development from pluripotency. *Nature*. **546**, 533-538(2017).

11 Ashburner, M. et al. Gene ontology: tool for the unification of biology. The Gene Ontology Consortium. *Nature Genet.* **25**, 25-29(2000).

12 Kanehisa, M. & Goto, S. KEGG: kyoto encyclopedia of genes and genomes. *Nucleic Acids Res.* **28**, 27-30(2000).

13 La Manno, G. et al. RNA velocity of single cells. *Nature*. **560**, 494-498(2018).

14 Trapnell, C. et al. The dynamics and regulators of cell fate decisions are revealed by pseudotemporal ordering of single cells. *Nat. Biotechnol.* **32**, 381-386(2014).

15 Qiu, X. et al. Single-cell mRNA quantification and differential analysis with Census. *Nat. Methods*. **14**, 309-315(2017).

16 Efremova, M., Vento-Tormo, M., Teichmann, S. A. & Vento-Tormo, R. CellPhoneDB: inferring cell-cell communication from combined expression of multi-subunit ligand-receptor complexes. *Nat. Protoc.* **15**, 1484-1506(2020).

17 Browaeys, R., Saelens, W. & Saeys, Y. NicheNet: modeling intercellular communication by linking ligands to target genes. *Nat. Methods*. **17**, 159-162(2020).

Abbreviations

AIM, apoptosis inhibitor of macrophage; ALB, albumin; ALT, alanine aminotransferase; AST, aspartate amino transferase; BEAM, branched expression analysis modeling; BMDM, bone marrow-derived macrophage; BMI, body mass index; BSA, bovine serum albumin; CCL4, c-c motif chemokine ligand 4; CT, computerized tomography; DC, dendritic cell, DEG, differentially expressed gene; EDTA, ethylenediaminetetraacetic acid; EGVB, esophagogastric variceal bleeding; FC, fold change; FDR, false discovery rate; GEM, gel beads in-emulsion; GLM, generalized linear model; GO, gene ontology; H&E, hematoxylin and eosin; HRP, horseradish peroxidase; HSC, hepatic stellate cell; HVPG, hepatic venous pressure gradient; ICG-R15, indocyanine green retention test after 15 min; INR, international normalized ratio; KC, kupffer cell; KEGG, kyoto encyclopedia of genes and genomes; MELD, model for end-stage liver disease score; mIHC, multiplex immunohistochemistry; MP, mononuclear-phagocyte; NAMPT, Nicotinamide Phosphoribosyltransferase; NC, negative control; NK, natural killer cell; OE, overexpression; PBS, phosphate buffered saline; PCA, principal component analysis; PCR, polymerase chain reaction; PLT, platelet count; PT, prothrombin time; RAM, regeneration-associated macrophages; RBC, red blood cell count; SEM, standard error of mean; Single-cell sequencing, scRNA-seq; TBIL, total bilirubin; TBST, tris buffered saline with tween; TSA, tyramide signal amplification technology; t-SNE, t-distributed stochastic neighbor embedding; UMAP, uniform manifold approximation and projection; UMI, unique

molecular identifier; WBC, white blood cell count.
